# Large Language Models’ Clinical Decision-Making on When to Perform a Kidney Biopsy: Comparative Study

**DOI:** 10.2196/73603

**Published:** 2025-09-18

**Authors:** Michael Toal, Christopher Hill, Michael Quinn, Ciaran O'Neill, Alexander P Maxwell

**Affiliations:** 1 Centre for Public Health, Royal Victoria Hospital, Queen’s University Belfast, Grosvenor Road, Belfast, BT12 6BA, United Kingdom, 44 28 9097 6350; 2 Regional Centre for Nephrology and Transplantation, Belfast City Hospital, Belfast, United Kingdom

**Keywords:** kidney biopsy, renal biopsy, nephrology, chronic kidney disease, kidney failure, proteinuria, hematuria, glomerulonephritis, machine learning, large language models, artificial intelligence, decision support

## Abstract

**Background:**

Artificial intelligence (AI) and large language models (LLMs) are increasing in sophistication and are being integrated into many disciplines. The potential for LLMs to augment clinical decision-making is an evolving area of research.

**Objective:**

This study compared the responses of over 1000 kidney specialist physicians (nephrologists) with the outputs of commonly used LLMs using a questionnaire determining when a kidney biopsy should be performed.

**Methods:**

This research group completed a large online questionnaire for nephrologists to determine when a kidney biopsy should be performed. The questionnaire was co-designed with patient input, refined through multiple iterations, and piloted locally before international dissemination. It was the largest international study in the field and demonstrated variation among human clinicians in biopsy propensity relating to human factors such as sex and age, as well as systemic factors such as country, job seniority, and technical proficiency. The same questions were put to both human doctors and LLMs in an identical order in a single session. Eight commonly used LLMs were interrogated: ChatGPT-3.5, Mistral Hugging Face, Perplexity, Microsoft Copilot, Llama 2, GPT-4, MedLM, and Claude 3. The most common response given by clinicians (human mode) for each question was taken as the baseline for comparison. Questionnaire responses on the indications and contraindications for biopsy generated a score (0-44) reflecting biopsy propensity, in which a higher score was used as a surrogate marker for an increased tolerance of potential associated risks.

**Results:**

The ability of LLMs to reproduce human expert consensus varied widely with some models demonstrating a balanced approach to risk in a similar manner to humans, while other models reported outputs at either end of the spectrum for risk tolerance. In terms of agreement with the human mode, ChatGPT-3.5 and GPT-4 (OpenAI) had the highest levels of alignment, agreeing with the human mode on 6 out of 11 questions. The total biopsy propensity score generated from the human mode was 23 out of 44. Both OpenAI models produced similar propensity scores between 22 and 24. However, Llama 2 and MS Copilot also scored within this range but with poorer response alignment to the human consensus at only 2 out of 11 questions. The most risk-averse model in this study was MedLM, with a propensity score of 11, and the least risk-averse model was Claude 3, with a score of 34.

**Conclusions:**

The outputs of LLMs demonstrated a modest ability to replicate human clinical decision-making in this study; however, performance varied widely between LLM models. Questions with more uniform human responses produced LLM outputs with higher alignment, whereas questions with lower human consensus showed poorer output alignment. This may limit the practical use of LLMs in real-world clinical practice.

## Introduction

### Artificial Intelligence in Health Care

The rapid expansion of artificial intelligence (AI) has impacted numerous disciplines over recent decades. This technology aims to improve efficiency; however, there are concerns that human roles may be replaced and that autonomous AI could cause significant disruption to societies [1,2]. AI is now seamlessly integrated into everyday life, and common functions such as predictive texting, review summaries, and customer service chatbots rely on this technology. Generative AI can be used to rapidly synthesize and assist with the creation of images and text, which has led academic institutions to consider how to effectively undertake assessments. Large language models (LLMs), such as ChatGPT, Copilot, and Llama, have rapidly proliferated to employ these developments for personal or professional use.

AI has also been used effectively in health care, and further expansion is predicted in the years ahead [3,4]. There are many demands on health care resources across the world [5], and AI offers opportunities to automate routine human tasks, allowing human practitioners to use their time more effectively on complex problems that are currently beyond the scope of AI [6,7]. In some circumstances, chatbot-generated outputs have even been found to be of higher quality and convey deeper empathy than human responses [8,9]. Diagnostic specialties offer a good template for this illustration. In pathology, AI has been used to rapidly characterize the nature of lesions for rapid detection and coding, allowing the pathologist to analyze specimens with greater efficiency [10]. In radiology, similar pattern recognition has been used to quickly identify abnormalities, as well as help the radiologist prioritize their workflow, so that the most abnormal or urgent scans are reported first [7].

### Limitations of Artificial Intelligence

The rise in AI usage has raised significant concerns. Intelligence is not equivalent to wisdom, and AI outputs are dependent on the data used to train these models. Although Generative AI can produce a detailed response, one criticism is that its output lacks the “common sense” of humans [11]. LLMs can generate false information in the form of hallucinations and produce gender- or racially biased outputs [2,12,13]. LLMs are sensitive to phrasing and can generate errors by varying the order of words [14]. How each company trains its LLMs remains confidential, and this lack of transparency is another cause of concern [3]. Using AI within health care is dependent on its alignment with human values to establish trust from service users, which is another challenge of any new technology, especially given the issues discussed [15].

### Clinical Decision Support

Medical practitioners make numerous clinical decisions throughout their working day. How they make these decisions remains poorly understood and open to many potential biases and influences [16,17]. Given that some of these decisions may stand between life and death, harnessing AI to assist physicians in making the best clinical decisions for each patient based on the available body of evidence may represent an opportunity to improve efficiency and enhance safe patient care. LLM outputs have been found to be superior to junior surgical residents’ clinical decision-making but inferior to that of senior colleagues; however, in these studies, LLM outputs were limited by inconsistencies and inaccuracies [18,19]. LLMs have been shown to easily pass high-stakes written medical examinations such as the United States Medical Licensing Examination (USMLE) [20] and the Membership of the Royal College of Physicians of the United Kingdom (MRCP(UK)) [21]; however, they appear to perform poorly in questions related to rare diseases, perhaps due to a paucity of training data [22]. In the Polish nephrology specialty examination, GPT-4 performed at a level similar to the average human candidate but below that of the top candidates [23].

Our research group completed a large international survey of physicians’ clinical decision-making, recruiting over 1000 doctors from 83 countries to complete a short online questionnaire [24]. In this study, nephrologists (kidney specialists) were asked to determine when a kidney biopsy was required using clinical scenarios of potential indications and contraindications. A kidney biopsy is used to define the type of kidney disease a patient has so that appropriate treatment can be administered. A biopsy is an invasive investigation with a small but significant risk of serious bleeding complications [25]. The use of AI in nephrology is increasing with recent studies assessing LLM usage for guideline adherence, dialysis management, and specialist examinations [26-29].

We aimed to compare the responses of over 1000 human doctors with those of LLMs, using the same questions on biopsy practice in the same order, to determine if AI can be used as a clinical decision tool in a safe and effective manner.

## Methods

### Questionnaire Design

The detailed methods for the questionnaire have been described in a previous paper [24]. In brief, the questionnaire was designed for kidney specialist doctors (nephrologists) to investigate the indications and contraindications for a kidney biopsy. The research team, consisting of 4 nephrologists and 1 health economist, co-designed the questionnaire with input from patient participants. This instrument was refined through multiple iterations and a pilot study undertaken in a group of 39 clinicians before wider dissemination. A biopsy propensity score between 0‐44 was generated based on the responses to 11 questions (0‐4) on indications and contraindications, with a higher score demonstrating an increased propensity to recommend biopsy in a given scenario and therefore a greater tolerance of the associated risks. Scores of 0‐44 were normalized to 0%‐100% for clarity. This allowed comparisons between nephrologists to determine if they were more or less likely to recommend this investigation when placed in an identical clinical situation. For each question, respondents were asked to select 1 of 5 possible responses to the prompt. For the clinical vignettes on indications, this was on a Likert scale from “definitely yes” to “definitely no,” and for contraindications, by defining a threshold of acceptable risk for clinical parameters associated with bleeding complications. The most common response (mode) for each question given by human respondents was determined to be the baseline for comparison with LLM outputs. For each question, the mode was selected by a minimum of 345 and a maximum of 728 human clinicians.

### LLM Application

Responses to the human questionnaire were collected from August 2023 to January 2024. LLMs were interrogated from March 2024 to June 2024. At this time, the results were not publicly available and therefore could not have been part of the evidence base used by the LLM to generate responses. The questions put to the LLM were identical (except for removing the words “in your opinion”) to those presented to human clinicians. They were also presented in the same order in a single session. The full transcripts generated by the LLMs are included in Multimedia Appendix 1 and were reviewed by the first author to assign scoring, which was subsequently reviewed and verified by two other coauthors (CON and APM).

A propensity score was generated for each LLM based on the responses to these 11 questions using the same scoring method as for human respondents. Therefore, an LLM that generated a higher score would be more inclined to recommend this investigation and therefore less risk averse. By contrast, a lower score would be indicative of being less inclined to recommend this investigation and more risk averse. An LLM was determined as being a perfect match to human clinicians if the answer selected was identical to the mode in the human questionnaire.

### Ethical Considerations

Ethical approval for this project was granted by the Faculty of Medicine, Health, and Life Sciences Research Ethics Committee of Queen’s University, Belfast (project MHLS 22_175) on February 15, 2023, and was conducted in accordance with the Declaration of Helsinki. Human participants completed an online questionnaire about kidney biopsy practice [24]. A statement giving consent to participate was displayed to the clinician on the first screen of the questionnaire. No identifying information was collected. No compensation was provided to participants.

## Results

### Human Respondents’ Characteristics

A total of 1181 clinicians from 83 countries participated in the study. A summary of clinician characteristics is given in Table 1. The study was open to nephrologist trainees and fellows who comprised 14.3% (n=168) of the total cohort.

The United States has the largest single national group, and 43 states were represented in this cohort. The 4 devolved nations in the United Kingdom were also represented in the second largest cumulative group. Thirteen nations had more than 20 clinicians included.

**Table 1.** Characteristics of human participants.

Characteristics	Values, n (%)
Sex	
Male	753 (64.3)
Female	408 (34.8)
Prefer not to say	9 (0.8)
Nonbinary/third gender	2 (0.2)
Age (y)	
20‐29	30 (2.5)
30‐39	442 (37.5)
40‐49	327 (27.7)
50‐59	251 (21.3)
60 or older	130 (11.0)
Current job title	
Trainee/fellow	168 (14.3)
Associate specialist/specialty doctor	122 (10.4)
Consultant/attending physician	733 (62.2)
Clinical director or professor	154 (13.1)
Other	1 (0.1)
Continent of practice	
Europe	405 (34.4)
North America	352 (29.9)
South America	85 (7.2)
Asia	216 (18.3)
Africa	67 (5.7)
Oceania	54 (4.6)

### LLM Interrogation

A total of 8 LLMs were interrogated, as detailed in Table 2. The full transcripts of the dialogues are detailed in Multimedia Appendix 1. The outputs produced by the LLMs varied greatly in terms of detail; however, each LLM was instructed to choose an answer from 5 options. Some LLMs selected more than 1 answer to certain prompts, refused to give an answer, or produced incomplete sentences; however, in most instances, the question was answered as instructed. An introductory prompt for context was added for GPT-4. The programs that were free to use without subscription were interrogated by the first author. GPT-4 and MedLM were not freely available; therefore, an additional operator with access was employed to reproduce these methods.

**Table 2.** Large language models (LLMs) used and dates of interrogation.

LLM	Date of interrogation	Availability
OpenAI: ChatGPT-3.5	March 27, 2024	Free without subscription
Mistral Hugging Face	March 28, 2024	Free without subscription
Perplexity	March 28, 2024	Free without subscription
Microsoft Copilot	March 28, 2024	Free without subscription
Llama 2 13b chatbot	April 3, 2024	Free without subscription
OpenAI: GPT-4	April 22, 2024	Subscription
MedLM	April 26, 2024	Subscription
Claude 3	June 13, 2024	Free without subscription

### LLM Prompts

Clinicians were asked whether, in their opinion, a kidney biopsy was required in the setting of 7 fictional clinical vignettes. All cases were adults with unexplained abnormalities in kidney function, reported as estimated glomerular filtration rate and urinary tests (hematuria or proteinuria quantified as grams per day). Four cases were a first presentation to a nephrologist, and in three, there was a dynamic change over the course of a year.

The determination of when clinicians felt the risk of kidney biopsy outweighed the benefits was explored in a section on potential contraindications, particularly relating to bleeding risk. In the first section, clinicians were presented with 5 options and asked for the limits of acceptable parameters to proceed to biopsy. This could be the minimum level (eg, hemoglobin) or maximum level (eg, systolic blood pressure). The question prompts given to the LLMs are detailed in Table 3.

**Table 3.** Question prompts given to large language models.

Question code	Full question
Q1	Is a renal biopsy required for an adult in the first detection of an unexplained nephrotic syndrome of proteinuria 4g/day, peripheral oedema and eGFR^a^>60 ml/min/1.73m^2^? Choose from Definitely yes, Probably yes, Unsure, Probably not, Definitely not
Q2	Is a renal biopsy required for an adult in the first detection of unexplained non-visible haematuria, 2g/day of proteinuria and eGFR 40? Choose from Definitely yes, Probably yes, Unsure, Probably not, Definitely not
Q3	Is a renal biopsy required for an adult in the first detection of unexplained non-visible haematuria, 2g/day of proteinuria and eGFR 20 with normal kidney appearances on ultrasound? Choose from Definitely yes, Probably yes, Unsure, Probably not, Definitely not
Q4	Is a renal biopsy required for an adult in the first detection of unexplained non-visible haematuria, 2g/day of proteinuria and eGFR 20 with reduced kidney size on ultrasound? Choose from Definitely yes, Probably yes, Unsure, Probably not, Definitely not
Q5	Is a renal biopsy required for an adult with an unexplained rise in proteinuria from 0.5 to 2g/day in one year with an eGFR>60? Choose from Definitely yes, Probably yes, Unsure, Probably not, Definitely not
Q6	Is a renal biopsy required for an adult with an unexplained fall in eGFR from 55 to 40 in one year with proteinuria stable at 0.5g/day? Choose from Definitely yes, Probably yes, Unsure, Probably not, Definitely not
Q7	Is a renal biopsy required for an adult with an unexplained fall in eGFR from 55 to 40 AND rise in proteinuria from 0.5 to 2 g/day in one year? Choose from Definitely yes, Probably yes, Unsure, Probably not, Definitely not
Q8	What is the minimum acceptable Haemoglobin for native renal biopsy? Choose from 100g/l, 90 g/l, 80 g/l, Other (please specify) and No minimum level
Q9	What is the minimum acceptable Platelet count for native renal biopsy? Choose from 150×109, 100×109, 50×109, Other (please specify), No minimum level
Q10	What is the maximum acceptable International Normalised Ratio for native renal biopsy? Choose from 1.2, 1.4, 1.6, Other (please specify) and No maximum level
Q11	What is the maximum acceptable Systolic Blood Pressure for native renal biopsy? Choose from 140 mmHg, 160 mmHg, 180 mmHg, Other (please specify) and No maximum level

a eGFR: estimated glomerular filtration rate.

### Comparing Human Doctor and LLM Responses

In the human questionnaire, all available options were selected by at least 3 and at most 728 human doctors. Therefore, none of the LLM outputs could be considered outside the range of responses that a human doctor may select. The subject of kidney biopsy decision-making was chosen for this clinician questionnaire because it is subjective; therefore, there is a range of acceptable answers. The mode of each answer represents varying proportions of responses to each question. For question 8, there was no clear consensus, and the mode was selected by only 31.6% of respondents; however, for question 9, the consensus was clearer, and 66.5% of respondents selected the mode. Responses are detailed in Table 4.

The level of agreement between the mode of human responses and LLM outputs ranged from 0 out of 11 (Mistral Hugging Face) to 6 out of 11 (ChatGPT-3.5 and GPT-4). Four of eight LLMs generated a biopsy propensity score that was equal to or within one point of the human mode score (ChatGPT-3.5, GPT-4, MS CoPilot, and Llama 2).

Using this propensity score as a surrogate marker for clinical risk aversion, the most risk-averse LLM output was MedLM with a score of 11, which produced outputs equivalent to the lowest 1% of biopsy propensity scores in human respondents. By contrast, the Claude 3 output produced the highest biopsy propensity score of 34, indicating the lowest level of risk aversion, a score higher than 99% of human respondents. For both MedLM and Claude 3, there was a reasonable agreement between outputs and human responses with 4 or 5 exact matches out of 11; however, the overall approach to risk, as indicated by the propensity score, was not typical of human responses.

In terms of which LLM most accurately represented human doctor responses, the two OpenAI LLMs, ChatGPT-3.5 and GPT-4, were the optimal programs for agreement with the human mode and profile of risk aversion, as indicated by the propensity score.

**Table 4.** Comparison of questionnaire responses between human consensus and large language models (LLMs).

Question	Humans (N=1181)^a^, n (%)^b^	ChatGPT3.5	Mistral Hugging Face	Perplexity	MS Copilot	Llama 2 13b chat	GPT4	MedLM	Claude 3
Q1	DY^c^, 655 (58.1)	PY^d^	NA^e^	DY^f^	PY	U^g^	DY^f^ and PY	PY	DY^f^
Q2	DY, 659 (59)	DY^f^	NA	DY^f^	PY	PY	PY	PN^h^	PY
Q3	DY, 571 (51*.*2)	DY^f^	NA	DY^f^	PY	U	DY^f^	PN	DY^f^
Q4	PN, 571 (51.4)	DY	NA	DY	PY	DY	PN^f^ and DN^i^	PN^f^	DY
Q5	PY, 521 (47.0)	PN	NA	PY^f^	U	PY^f^	PY^f^	PN	PY^f^
Q6	PN, 398 (36.1)	PN^f^	NA	PY	PY	U	U	PN^f^	PY
Q7	PY, 565 (51.7)	PY^f^	NA	DY	PY^f^	U and PY^f^	DY	PY^f^	DY
Q8 Hb^j^ (g/l)	90, 345 (31.6)	100	80	Other	Other	80	100	90^f^	80
Q9 Plat^k^ (×10^9^)	100, 728 (66.5)	100^f^	50	50	100^f^	150	100^f^	100^f^	50
Q10 INR^l^	1.2, 557 (51.1)	1.4	1.4	1.5	1.5	1.4	1.4	1.4	1.5
Q11 SBP^m^ (mmHg)	160, 600 (54.7)	160^f^	140	140	140	140	160^f^	140	160^f^
Total score	23	23	—^n^	29	23	22	22‐24	11	34
Agreement	—	6/11	0/11	4/11	2/11	2/11	6/11	5/11	4/11

a There are small variations in the numbers of human participants who answered each question, and the denominator of the percentage is derived from the number of humans who answered each question, rather than total participants in the study. More details are provided on this in the following study [24].

b Most common response (mode) given by human participants. Numbers in parentheses represent the proportion of human respondents who selected the mode for each question.

c DY: definitely yes.

d PY: probably yes.

e NA: no answer given.

f LLM output contains mode of human responses.

g U: unsure.

h PN: probably not.

i DN: definitely not.

j Hb: hemoglobin.

k Plat: platelet count.

l INR: international normalized ratio.

m SBP: systolic blood pressure.

n Not applicable.

## Discussion

### Principal Results

In this study, the questionnaire responses of nephrologists on clinical decision-making were replicated by some LLMs. The degree of fidelity differed among LLMs, and the OpenAI models ChatGPT-3.5 and GPT-4 produced outputs that were the most consistent with typical clinician responses. Similar to our study of human clinicians, most of the LLMs interrogated opted for a balanced approach to this dilemma, producing comparable responses about when to perform a kidney biopsy and when to avoid this procedure.

There were varying degrees of agreement among human respondents, with the mode selected by 31.6% to 66.5% of respondents. This variation brings ambiguity to the “ground-truth” in each scenario with inconsistent dispersion of answers. This spectrum of consensus was also replicated among LLMs. In question 8, where only 31.6% of human respondents selected the mode, the mode was selected by only 1 out of 8 LLMs, the lowest in our study. Conversely, in question 9 with the highest agreement, where 66.5% of humans selected the mode, this human mode was also selected by 4 out of 8 LLMs, the joint highest in our study. This suggests that the gray areas of ambiguity in clinical decision-making can also be reflected in the LLM outputs. One potential use for this technology would be to assist the clinician in resolving an uncertain decision; however, in this instance, this uncertainty is also reflected in LLM outputs, limiting their utility in real-world clinical practice.

The propensity to perform an invasive kidney biopsy procedure is inevitably linked to tolerance of potential risks. Therefore, we used the propensity score as a surrogate marker for risk aversion among human clinicians. When this score was applied to LLMs, there was variable risk aversion among these models. MedLM outputs were the most risk-averse, indicating a higher threshold to perform a kidney biopsy, as well as a low tolerance for potential contraindications that would increase the risk of a bleeding complication. In contrast, the outputs for Claude 3 were the least risk-averse, meaning every clinical vignette was met with a response that a kidney biopsy was definitely or probably required, and the lower limits for potential contraindications could be considered by some clinicians to be reckless.

The length and detail of outputs generated by each LLM were variable, as described in Multimedia Appendix 1. Some models such as ChatGPT-3.5 and MedLM answered the question directly, with limited additional discussion of the reasoning behind decisions. Other models such as Llama 2 and Microsoft Copilot produced detailed responses outlining the dilemma and the known variances in practice before reaching a conclusion. The updated OpenAI model GPT-4 produced much longer outputs based on mean word count (164 words) compared to its predecessor ChatGPT-3.5 (19 words).

AI applications are currently in development to analyze large volumes of free text to allow for organized coding of data for research and analytic purposes [6]. LLMs are improving rapidly, and specialized medical LLMs have demonstrated significant improvement with continual pretraining and instructional fine-tuning for tasks such as question answering, summarization, disease classification, and natural language inference [30]. A US study compared human and LLM capabilities in detecting adverse events from a cannabinoid-based product from posts on a social media group, using human evaluators as a benchmark. In this study, ChatGPT-3.5 was able to detect any adverse events with 95% agreement with humans, and 99% agreement for serious events [31]. However, LLMs are not sufficiently reliable for clinical care, as using AI scribes for physicians’ notes has produced text with significant errors, both by omission and by the inclusion of false statements [32].

### Limitations

This study has several limitations that should be considered. First, this is a small sample of 11 questions used to interrogate LLMs; therefore, there is limited depth to this data, and caution is required not to overinterpret the reported results. Second, this study assessed the LLMs’ ability to make decisions based on short, simple case vignettes, and this may not necessarily be generalizable to more nuanced and complex “real-life” clinical scenarios, as LLM accuracy has been shown to be poorer on longer questions [21]. Third, using a mode as the human benchmark is a limitation for questions with poor consensus, where the “ground-truth” is less evident; moreover, all human responses were treated as equal, despite vastly differing levels of clinical experience.

### Strengths

This study also has notable strengths. Human decision-making is poorly understood, and clinical decisions should be based on integrating the best available evidence for the care of an individual patient. AI-assisted decision aids are rapidly expanding into medicine, and this is the first study to our knowledge that compares a large sample of human responses to LLM outputs based on identical scenarios.

### Implications for Future Research

There has been a rapid proliferation of medical research into the use of AI in health care; however, how these tools are best integrated into clinical practice remains unclear. As LLMs continue to increase in sophistication and accuracy, AI assistance will likely become integral to all aspects of life. How best to apply this technology in health care remains a challenge to be addressed in the coming years. It is important that LLM outputs align with human values, which can be achieved through supervised reinforcement learning with input from expert physicians and patients [15].

### Conclusions

Some LLMs can modestly replicate human clinical decision-making when short clinical vignettes are presented. There is variable performance in these models; however, ChatGPT-3.5 and GPT-4 outputs were the most consistent with humans in our study. Caution should be applied when considering how these LLMs can be used to assist clinicians, as there remain many unanswered questions as to how physicians should use these tools for safe and effective patient care.

## Supplementary material

10.2196/73603 Multimedia Appendix 1 Transcript of large language model responses.

## Abbreviations

AI: artificial intelligence
LLM: large language model
MRCP(UK): Membership of the Royal College of Physicians
USMLE: United States Medical Licensing Examination
