# Supplementary material for: Large Language Models’ Clinical Decision-Making on When to Perform a Kidney Biopsy: Comparative Study
Source: J Med Internet Res. 2025 Sep 18;27:e73603. doi: 10.2196/73603 (PMC12445783; doi:10.2196/73603)
Supplement: Multimedia Appendix 1 [file jmir-v27-e73603-s001.docx]

Large Language Models’ Clinical Decision-Making on When to Perform a Kidney Biopsy: Comparative Study

Supplement

Contents

[Large language model transcripts 1](#_Toc188439759)

[Chat GPT 3.5 1](#_Toc188439760)

[Mistral on hugging face 4](#_Toc188439761)

[Perplexity 6](#_Toc188439762)

[MS Co-pilot 12](#_Toc188439763)

[Llama 2 chat 13b 16](#_Toc188439764)

[Chat GPT 4.0 22](#_Toc188439765)

[MedLM 33](#_Toc188439766)

[Claude 3 Sonnet 35](#_Toc188439767)

# Large language model transcripts

## Chat GPT 3.5

27.3.24

Chat AI GPT:

Hello human, I am a GPT powered AI chat bot. Ask me anything!

You:

Is a renal biopsy required for an adult in the first detection of an unexplained nephrotic syndrome of proteinuria 4g/day, peripheral oedema and eGFR>60 ml/min/1.73m2? Choose from Definitely yes, Probably yes, Unsure, Probably not, Definitely not

Chat AI GPT:

Based on the provided context, the most appropriate response would be "Probably yes." A renal biopsy may be necessary in this situation to further investigate the underlying cause of the unexplained nephrotic syndrome, especially with significant proteinuria and peripheral edema. It would help in determining the specific type of kidney disease and guiding appropriate treatment.

You:

Is a renal biopsy required for an adult in the first detection of unexplained non-visible haematuria, 2g/day of proteinuria and eGFR 40? Choose from Definitely yes, Probably yes, Unsure, Probably not, Definitely not

Chat AI GPT:

Definitely yes. A renal biopsy may be necessary for an adult with unexplained non-visible hematuria, 2g/day of proteinuria, and an eGFR of 40. It can help determine the underlying cause of these symptoms and guide appropriate treatment.

You:

Is a renal biopsy required for an adult in the first detection of unexplained non-visible haematuria, 2g/day of proteinuria and eGFR 20 with normal kidney appearances on ultrasound? Choose from Definitely yes, Probably yes, Unsure, Probably not, Definitely not

Chat AI GPT:

Definitely yes.

You:

is a renal biopsy required for an adult in the first detection of unexplained non-visible haematuria, 2g/day of proteinuria and eGFR 20 with reduced kidney size on ultrasound? Choose from Definitely yes, Probably yes, Unsure, Probably not, Definitely not

Chat AI GPT:

Definitely yes. A renal biopsy may be required for an adult in the first detection of unexplained non-visible hematuria, 2g/day of proteinuria, eGFR 20, and reduced kidney size on ultrasound. It is important to further investigate the underlying cause of these symptoms to determine the appropriate treatment plan.

You:

is a renal biopsy required for an adult with an unexplained rise in proteinuria from 0.5 to 2g/day in one year with an eGFR > 60? Choose from Definitely yes, Probably yes, Unsure, Probably not, Definitely not

Chat AI GPT:

Probably not.

You:

is a renal biopsy required for an adult with an unexplained fall in eGFR from 55 to 40 in one year with proteinuria stable at 0.5g/day? Choose from Definitely yes, Probably yes, Unsure, Probably not, Definitely not

Chat AI GPT:

Probably not.

You:

is a renal biopsy required for an adult with an unexplained fall in eGFR from 55 to 40 AND rise in proteinuria from 0.5 to 2 g/day in one year? Choose from Definitely yes, Probably yes, Unsure, Probably not, Definitely not

Chat AI GPT:

Probably yes.

You:

What is the minimum acceptable Haemogloblin for native renal biopsy? Choose from 100g/l, 90 g/l, 80 g/l, Other (please specify) and No minimum level

Chat AI GPT:

The minimum acceptable Hemoglobin level for a native renal biopsy is typically around 100g/l.

You:

What is the minimum acceptable Platelet count for native renal biopsy? Choose from 150 x10^9^, 100 x10^9^, 50 x10^9^, Other (please specify), No minimum level

Chat AI GPT:

The minimum acceptable Platelet count for a native renal biopsy is typically around 100 x 10^9.

You:

What is the maximum acceptable International Normalised Ratio for native renal biopsy? Choose from 1.2, 1.4, 1.6, Other (please specify) and No maximum level

Chat AI GPT:

The maximum acceptable International Normalised Ratio for a native renal biopsy is typically around 1.4.

You:

What is the maximum acceptable Systolic Blood Pressure for native renal biopsy? Choose from 140 mmHg, 160 mmHg, 180 mmHg, Other (please specify) and No maximum level

Chat AI GPT:

The maximum acceptable Systolic Blood Pressure for a native renal biopsy is typically around 160 mmHg.

## Mistral on hugging face

28.3.24

Q1: Is a renal biopsy required for an adult in the first detection of an unexplained nephrotic syndrome of proteinuria 4g/day, peripheral oedema and eGFR>60 ml/min/1.73m2? Choose from Definitely yes, Probably yes, Unsure, Probably not, Definitely not

A1: The decision to perform a renal biopsy in an adult with an unexplained nephrotic syndrome (proteinuria >4g/day, peripheral edema) and an estimated glomerular filtration rate (eGFR) >60 ml/min/1.73m2 depends on various factors and should be made in consultation with a nephrologist. Nephrotic syndrome is a clinical syndrome characterized by heavy protein

Q2: is a renal biopsy required for an adult in the first detection of unexplained non-visible haematuria, 2g/day of proteinuria and eGFR 40? Choose from Definitely yes, Probably yes, Unsure, Probably not, Definitely not

A2 The decision to perform a renal biopsy in an adult with the first detection of unexplained non-visible hematuria (blood in urine that is not visible to the naked eye), 2g/day of proteinuria, and an estimated glomerular filtration rate (eGFR) of 40 ml/min/1.73m2 is more complex and should be made in consultation with a nephrologist.

Q3: Is a renal biopsy required for an adult in the first detection of unexplained non-visible haematuria, 2g/day of proteinuria and eGFR 20 with normal kidney appearances on ultrasound? Choose from Definitely yes, Probably yes, Unsure, Probably not, Definitely not

A3: The decision to perform a renal biopsy in an adult with the first detection of unexplained non-visible hematuria (blood in urine that is not visible to the naked eye), 2g/day of proteinuria, and an estimated glomerular filtration rate (eGFR) of 20 ml/min/1.73m2 with normal kidney appearances on ultrasound is more complex and should be made in

Q4: is a renal biopsy required for an adult in the first detection of unexplained non-visible haematuria, 2g/day of proteinuria and eGFR 20 with reduced kidney size on ultrasound? Choose from Definitely yes, Probably yes, Unsure, Probably not, Definitely not

A4: The decision to perform a renal biopsy in an adult with the first detection of unexplained non-visible hematuria (blood in urine that is not visible to the naked eye), 2g/day of proteinuria, and an estimated glomerular filtration rate (eGFR) of 20 ml/min/1.73m2 with reduced kidney size on ultrasound is more complex and should be made in

Q5: is a renal biopsy required for an adult with an unexplained rise in proteinuria from 0.5 to 2g/day in one year with an eGFR > 60? Choose from Definitely yes, Probably yes, Unsure, Probably not, Definitely not

A5: The decision to perform a renal biopsy in an adult with an unexplained rise in proteinuria from 0.5 to 2g/day in one year and an estimated glomerular filtration rate (eGFR) >60 ml/min/1.73m2 depends on various factors and should be made in consultation with a nephrologist. A rise in proteinuria, particularly if it is significant and unex

Q6: is a renal biopsy required for an adult with an unexplained fall in eGFR from 55 to 40 in one year with proteinuria stable at 0.5g/day? Choose from Definitely yes, Probably yes, Unsure, Probably not, Definitely not

A6: The decision to perform a renal biopsy in an adult with an unexplained fall in estimated glomerular filtration rate (eGFR) from 55 to 40 ml/min/1.73m2 in one year and stable proteinuria of 0.5g/day depends on various factors and should be made in consultation with a nephrologist. A decrease in eGFR, particularly if it is significant and

Q7: is a renal biopsy required for an adult with an unexplained fall in eGFR from 55 to 40 AND rise in proteinuria from 0.5 to 2 g/day in one year? Choose from Definitely yes, Probably yes, Unsure, Probably not, Definitely not

A7: The decision to perform a renal biopsy in an adult with an unexplained fall in estimated glomerular filtration rate (eGFR) from 55 to 40 ml/min/1.73m2 and a rise in proteinuria from 0.5 to 2g/day in one year depends on various factors and should be made in consultation with a nephrologist. Both a decrease in eGFR

Q: What is the minimum acceptable Haemogloblin for native renal biopsy? Choose from 100g/l, 90 g/l, 80 g/l, Other (please specify) and No minimum level

A: The minimum acceptable hemoglobin (Hb) level for a native renal biopsy can depend on the specific guidelines of the healthcare institution or the preferences of the nephrologist or radiologist performing the biopsy. However, most guidelines recommend that the hemoglobin level be above 80 g/L or ideally above 100 g/L for a safe and effective biopsy. Some centers may consider performing the biopsy with

Q: What is the minimum acceptable Platelet count for native renal biopsy? Choose from 150 x109, 100 x109, 50 x109, Other (please specify), No minimum level

A: The minimum acceptable platelet count for a native renal biopsy can depend on the specific guidelines of the healthcare institution or the preferences of the nephrologist or radiologist performing the biopsy. However, most guidelines recommend that the platelet count be above 50 x 10^9/L or ideally above 100 x 10^9/L for a safe and effective biopsy. Some centers may consider performing the biopsy with

Q: What is the maximum acceptable International Normalised Ratio for native renal biopsy? Choose from 1.2, 1.4, 1.6, Other (please specify) and No maximum level

A: The maximum acceptable international normalized ratio (INR) for a native renal biopsy can depend on the specific guidelines of the healthcare institution or the preferences of the nephrologist or radiologist performing the biopsy. However, most guidelines recommend that the INR be below 1.4 or ideally below 1.2 for a safe and effective biopsy. Some centers may consider performing the biopsy with a higher INR, but this would depend on the specific

Q: What is the maximum acceptable Systolic Blood Pressure for native renal biopsy? Choose from 140 mmHg, 160 mmHg, 180 mmHg, Other (please specify) and No maximum level

A: The maximum acceptable systolic blood pressure (BP) for a native renal biopsy can depend on the specific guidelines of the healthcare institution or the preferences of the nephrologist or radiologist performing the biopsy. However, most guidelines recommend that the systolic BP be below 140 mmHg or ideally below 130 mmHg for a safe and effective biopsy. Some centers may consider performing the biopsy with a systolic B

## Perplexity

28.3.24

Q1: Is a renal biopsy required for an adult in the first detection of an unexplained nephrotic syndrome of proteinuria 4g/day, peripheral oedema and eGFR>60 ml/min/1.73m2? Choose from Definitely yes, Probably yes, Unsure, Probably not, Definitely not

A1: Based on the search results provided, the answer is Definitely yes.

The search results indicate that a renal biopsy is a standard and essential procedure for the diagnosis and management of unexplained nephrotic syndrome in adults:

"All cases of nephrotic syndrome should be referred to renal to discuss the need for a renal biopsy."

5

"Primary indications of kidney biopsy are glomerular hematuria/proteinuria with or without renal dysfunction and unexplained Kidney biopsy is usually performed in prone position but in certain situations, supine and lateral positions may be required."

4

"Renal biopsy is typically performed by a radiologist under computed tomography (CT) or ultrasonographic guidance. However, a urologist can also perform a kidney biopsy during renal surgery. There are multiple indications to perform renal biopsy, including the following: Unexplained renal failure, Isolated non-nephrotic proteinuria, Isolated glomerular hematuria, Connective-tissue diseases (e.g., systemic lupus erythematosus)"

2

The search results make it clear that a renal biopsy is the standard of care for evaluating unexplained nephrotic syndrome in adults, in order to determine the underlying cause and guide appropriate treatment. Therefore, the answer is Definitely yes, a renal biopsy is required in this case.

Q2: is a renal biopsy required for an adult in the first detection of unexplained non-visible haematuria, 2g/day of proteinuria and eGFR 40? Choose from Definitely yes, Probably yes, Unsure, Probably not, Definitely not

A2: Based on the search results provided, the answer is Definitely yes, a renal biopsy is required for an adult in the first detection of unexplained non-visible haematuria, 2g/day of proteinuria, and eGFR 40 ml/min/1.73m2.

The key evidence from the search results is:

"Non-visible haematuria with markers of abnormal kidney function (such as increasing serum creatinine, reduced urine output, hypertension or proteinuria) has a variety of potential urological and nephrological causes. Regarding intrinsic kidney disease, non-visible haematuria with deranged kidney function often reflects glomerular inflammation (glomerulonephritis). These patients may be systemically unwell and, in the presence of an acute decline in their kidney function, will require close monitoring and follow-up. Kidney biopsy is a key component to diagnosis, management and prognosis, whereby the focus of treatment is the underlying glomerulonephritis."

"Significant proteinuria (ACR ≥30 mg/mmol or PCR ≥50 mg/mmol) in addition to haematuria raises the suspicion of intrinsic Glomerular haematuria with macroalbuminuria"

"Renal biopsy is typically performed by a radiologist under computed tomography (CT) or ultrasonographic guidance. However, a urologist can also perform a kidney biopsy during renal surgery. There are multiple indications to perform renal biopsy, including the following: Unexplained renal failure, Isolated non-nephrotic proteinuria, Isolated glomerular hematuria, Connective-tissue diseases (e.g., systemic lupus erythematosus)"

The search results clearly indicate that the combination of unexplained non-visible haematuria, significant proteinuria (2g/day), and reduced kidney function (eGFR 40 ml/min/1.73m2) warrants a renal biopsy to determine the underlying cause and guide appropriate treatment. Therefore, the answer is Definitely yes, a renal biopsy is required in this case.

Q3: Is a renal biopsy required for an adult in the first detection of unexplained non-visible haematuria, 2g/day of proteinuria and eGFR 20 with normal kidney appearances on ultrasound? Choose from Definitely yes, Probably yes, Unsure, Probably not, Definitely not

A3: Based on the search results provided, the answer is Definitely yes, a renal biopsy is required for an adult in the first detection of unexplained non-visible haematuria, 2g/day of proteinuria, and eGFR 20 ml/min/1.73m2 with normal kidney appearances on ultrasound.

The key evidence from the search results is:

"Non-visible haematuria with markers of abnormal kidney function (such as increasing serum creatinine, reduced urine output, hypertension or proteinuria) has a variety of potential urological and nephrological causes. Regarding intrinsic kidney disease, non-visible haematuria with deranged kidney function often reflects glomerular inflammation (glomerulonephritis). These patients may be systemically unwell and, in the presence of an acute decline in their kidney function, will require close monitoring and follow-up. Kidney biopsy is a key component to diagnosis, management and prognosis, whereby the focus of treatment is the underlying glomerulonephritis."

"Significant proteinuria (ACR ≥30 mg/mmol or PCR ≥50 mg/mmol) in addition to haematuria raises the suspicion of intrinsic Glomerular haematuria with macroalbuminuria"

"Renal biopsy is typically performed by a radiologist under computed tomography (CT) or ultrasonographic guidance. However, a urologist can also perform a kidney biopsy during renal surgery. There are multiple indications to perform renal biopsy, including the following: Unexplained renal failure, Isolated non-nephrotic proteinuria, Isolated glomerular hematuria, Connective-tissue diseases (e.g., systemic lupus erythematosus)"

The search results clearly indicate that the combination of unexplained non-visible haematuria, significant proteinuria (2g/day), and severely reduced kidney function (eGFR 20 ml/min/1.73m2) warrants a renal biopsy to determine the underlying cause and guide appropriate treatment, even with normal kidney appearances on ultrasound. Therefore, the answer is Definitely yes, a renal biopsy is required in this case.

Q4: Is a renal biopsy required for an adult in the first detection of unexplained non-visible haematuria, 2g/day of proteinuria and eGFR 20 with reduced kidney size on ultrasound? Choose from Definitely yes, Probably yes, Unsure, Probably not, Definitely not

A4: Based on the search results provided, the answer is Definitely yes, a renal biopsy is required for an adult in the first detection of unexplained non-visible haematuria, 2g/day of proteinuria, and eGFR 20 ml/min/1.73m2 with reduced kidney size on ultrasound.

The key evidence from the search results is:

"Non-visible haematuria with markers of abnormal kidney function (such as increasing serum creatinine, reduced urine output, hypertension or proteinuria) has a variety of potential urological and nephrological causes. Regarding intrinsic kidney disease, non-visible haematuria with deranged kidney function often reflects glomerular inflammation (glomerulonephritis). These patients may be systemically unwell and, in the presence of an acute decline in their kidney function, will require close monitoring and follow-up. Kidney biopsy is a key component to diagnosis, management and prognosis, whereby the focus of treatment is the underlying glomerulonephritis."

"Significant proteinuria (ACR ≥30 mg/mmol or PCR ≥50 mg/mmol) in addition to haematuria raises the suspicion of intrinsic Glomerular haematuria with macroalbuminuria"

"Renal biopsy is typically performed by a radiologist under computed tomography (CT) or ultrasonographic guidance. However, a urologist can also perform a kidney biopsy during renal surgery. There are multiple indications to perform renal biopsy, including the following: Unexplained renal failure, Isolated non-nephrotic proteinuria, Isolated glomerular hematuria, Connective-tissue diseases (e.g., systemic lupus erythematosus)"

The search results clearly indicate that the combination of unexplained non-visible haematuria, significant proteinuria (2g/day), severely reduced kidney function (eGFR 20 ml/min/1.73m2), and reduced kidney size on ultrasound warrants a renal biopsy to determine the underlying cause and guide appropriate treatment. Therefore, the answer is Definitely yes, a renal biopsy is required in this case.

Q5: is a renal biopsy required for an adult with an unexplained rise in proteinuria from 0.5 to 2g/day in one year with an eGFR > 60? Choose from Definitely yes, Probably yes, Unsure, Probably not, Definitely not

A5: Based on the information from the provided sources, the answer is Probably yes. While the rise in proteinuria from 0.5 to 2g/day in one year with an eGFR > 60 does not immediately indicate a need for a renal biopsy, it is essential to consider the context and individual patient factors before making a definitive decision.

The search results highlight the importance of proteinuria and eGFR levels in assessing renal health. Patients with both eGFR <30 mL/min/1.73 m2 and proteinuria ≥0.5 g/day are at a significantly higher risk of renal events, emphasizing the relevance of these parameters in predicting outcomes

Additionally, isolated hematuria can sometimes be an early sign of glomerulonephritis, prompting nephrologists to consider renal biopsy for a more accurate diagnosis

In this scenario, while the rise in proteinuria is notable, the eGFR being above 60 ml/min/1.73m2 suggests relatively preserved kidney function. However, given the potential implications of increasing proteinuria and the importance of monitoring renal health closely, it would be prudent to consider further evaluation, which may include close monitoring, additional tests, or consultation with a nephrologist to determine if a renal biopsy is necessary. Therefore, the answer is Probably yes.

Q6: Is a renal biopsy required for an adult with an unexplained fall in eGFR from 55 to 40 in one year with proteinuria stable at 0.5g/day? Choose from Definitely yes, Probably yes, Unsure, Probably not, Definitely not

A6: Based on the information provided in the search results, the answer is Probably yes, a renal biopsy is required for an adult with an unexplained fall in eGFR from 55 to 40 ml/min/1.73m2 in one year with proteinuria stable at 0.5g/day.

The key evidence from the search results is:

Patients with both eGFR <30 mL/min/1.73 m2 and proteinuria ≥0.5 g/day had a 26.7-fold higher risk of renal events compared to those with eGFR ≥60 mL/min/1.73 m2 and proteinuria <0.5 g/day.

The incidence of renal events increased with lower baseline eGFR and greater baseline proteinuria levels.

Renal biopsy is typically performed for indications such as unexplained renal failure and isolated non-nephrotic proteinuria.

While the proteinuria level of 0.5g/day is not in the nephrotic range, the unexplained decline in eGFR from 55 to 40 ml/min/1.73m2 over one year is concerning and warrants further evaluation. A renal biopsy could help identify the underlying cause of the declining kidney function and guide appropriate management.

Therefore, the answer is Probably yes, a renal biopsy is required in this case to determine the cause of the unexplained eGFR decline and guide treatment, even though the proteinuria is stable at 0.5g/day.

Q7: Is a renal biopsy required for an adult with an unexplained fall in eGFR from 55 to 40 AND rise in proteinuria from 0.5 to 2 g/day in one year? Choose from Definitely yes, Probably yes, Unsure, Probably not, Definitely not

A7: Based on the information provided in the search results, the answer is Definitely yes, a renal biopsy is required for an adult with an unexplained fall in eGFR from 55 to 40 ml/min/1.73m2 and a rise in proteinuria from 0.5 to 2 g/day in one year.

The key evidence from the search results is:

Patients with both eGFR <30 mL/min/1.73 m2 and proteinuria ≥0.5 g/day had a significantly higher risk of renal events compared to those with eGFR ≥60 mL/min/1.73 m2 and proteinuria <0.5 g/day

Lower eGFR levels and higher proteinuria levels at the time of renal biopsy were independently associated with a higher risk for renal events

The combination of a fall in eGFR and a rise in proteinuria can indicate significant renal dysfunction and increased risk for adverse renal outcomes, warranting further evaluation through a renal biopsy

Given the substantial changes in both eGFR and proteinuria levels over a relatively short period, a renal biopsy is crucial to determine the underlying cause of these changes and guide appropriate management. Therefore, the answer is Definitely yes, a renal biopsy is required in this case.

Q8: What is the minimum acceptable Haemogloblin for native renal biopsy? Choose from 100g/l, 90 g/l, 80 g/l, Other (please specify) and No minimum level

A: Based on the information provided in the search results, there does not appear to be a clearly defined minimum acceptable hemoglobin level for performing a native renal biopsy. The search results indicate the following:

The search results do not specify a minimum acceptable hemoglobin level for performing a native renal biopsy.

Patients undergoing renal biopsy for evaluation of acute kidney injury (AKI) had a lower mean hemoglobin of 10.4 ± 1.7 g/dL compared to 12.1 ± 2.1 g/dL in patients biopsied for other reasons. However, the biopsy was still performed in the AKI group despite the lower hemoglobin.

The search results mention that if the bleeding time was elevated, desmopressin acetate (DDAVP) was administered prior to the biopsy procedure. This suggests there may not be a strict minimum hemoglobin requirement, but rather the focus is on optimizing coagulation status before the procedure.

Therefore, based on the information provided, there does not appear to be a clearly defined minimum acceptable hemoglobin level for performing a native renal biopsy. The decision to proceed with the biopsy seems to be based on an assessment of the overall clinical picture and coagulation status, rather than a specific hemoglobin threshold. The answer is Other (no minimum level specified).

Q9: What is the minimum acceptable Platelet count for native renal biopsy? Choose from 150 x10^9^, 100 x10^9^, 50 x10^9^, Other (please specify), No minimum level

A: Based on the information provided in the search results, the minimum acceptable platelet count for performing a native renal biopsy is 50 x 10^9^/L.

The key evidence from the search results is:

"In patients with platelet levels < 80 to 100 × 103/μL, platelet transfusions can be used to bring platelet counts closer to normal if the bleeding time is prolonged."

"Percutaneous liver biopsies are considered safe when the platelet count is at least 50 to 60 x 109/L (British Society of Gastroenterologists (BSG) 2004, Rockey, et al 2009). Below this level transjugular liver biopsy (TJLB) should be considered."

"We suggest that prior to renal biopsy, the platelet count should be above 50 000/μL and the INR should be less than 1.5."

"Overall, our safety margins for kidney biopsies (thrombocyte count >80 g/L and INR <1.4) appear to be safe, and a lower threshold (e.g., thrombocyte count >50 g/L) may be acceptable."

The search results consistently indicate that a platelet count of at least 50 x 109/L is the minimum acceptable level for performing a native renal biopsy. Some sources suggest a higher threshold of 80-100 x 109/L, but 50 x 109/L appears to be the minimum acceptable level based on the information provided.

Therefore, the answer is 50 x 109/L.

Q10: What is the maximum acceptable International Normalised Ratio for native renal biopsy? Choose from 1.2, 1.4, 1.6, Other (please specify) and No maximum level

A10: Based on the information provided in the search results, the maximum acceptable International Normalized Ratio (INR) for performing a native renal biopsy is 1.5.

The key evidence from the search results is:

"We suggest that prior to renal biopsy, the platelet count should be above 50 000/μL and the INR should be less than 1.5."

"Overall, our safety margins for kidney biopsies (thrombocyte count >80 g/L and INR <1.4) appear to be safe, and a lower threshold (e.g., thrombocyte count >50 g/L) may be acceptable."

The search results do not mention any other specific INR thresholds for performing a native renal biopsy.

The search results consistently indicate that an INR less than 1.5 is the maximum acceptable level for safely performing a native renal biopsy. Some sources suggest an even lower threshold of INR less than 1.4, but 1.5 appears to be the widely accepted upper limit.

Therefore, the answer is 1.5.

Q11: What is the maximum acceptable Systolic Blood Pressure for native renal biopsy? Choose from 140 mmHg, 160 mmHg, 180 mmHg, Other (please specify) and No maximum level

A11: Based on the information provided in the search results, the maximum acceptable Systolic Blood Pressure for performing a native renal biopsy is 140 mmHg.

The key evidence from the search results is:

"Patients undergoing native renal biopsy who have an SBP greater than 140 mm Hg or DBP greater than 90 mm Hg are at higher risk for bleeding complications."

"Systolic blood pressure ≥ 140 mmHg (%), 3 (21.4), 85 (14.4), 0.5, 0.18-1.66"

The search results do not mention any other specific systolic blood pressure thresholds for performing a native renal biopsy.

The search results consistently indicate that a systolic blood pressure above 140 mmHg is associated with a higher risk of bleeding complications following a native renal biopsy. There is no mention of higher thresholds being acceptable.

Therefore, the answer is 140 mmHg.

## MS Co-pilot

28.3.24

Q1: Is a renal biopsy required for an adult in the first detection of an unexplained nephrotic syndrome of proteinuria 4g/day, peripheral oedema and eGFR>60 ml/min/1.73m2? Choose from Definitely yes, Probably yes, Unsure, Probably not, Definitely not

A1: Based on the information I found, a renal biopsy may be considered in cases of new-onset nephrotic syndrome in adults, evidence of proteinuria greater than 1–2 g/24 h with or without hypertension1. However, the decision to proceed with a renal biopsy can depend on various factors, including the patient’s overall health, the presence of other medical conditions, and the specific circumstances of the case2. Therefore, I would say the answer is Probably yes. It’s always best to consult with a healthcare professional who can provide guidance based on a comprehensive understanding of the situation. Please note that this information might be outdated or incomplete, and it’s always best to consult with a healthcare professional for the most accurate information.

Q2: is a renal biopsy required for an adult in the first detection of unexplained non-visible haematuria, 2g/day of proteinuria and eGFR 40? Choose from Definitely yes, Probably yes, Unsure, Probably not, Definitely not

A2: Based on the information I found, a renal biopsy may be considered in cases of unexplained non-visible haematuria, proteinuria of 2g/day, and an eGFR of 401234. However, the decision to proceed with a renal biopsy can depend on various factors, including the patient’s overall health, the presence of other medical conditions, and the specific circumstances of the case1. Therefore, I would say the answer is Probably yes. It’s always best to consult with a healthcare professional who can provide guidance based on a comprehensive understanding of the situation. Please note that this information might be outdated or incomplete, and it’s always best to consult with a healthcare professional for the most accurate information.

Q3: Is a renal biopsy required for an adult in the first detection of unexplained non-visible haematuria, 2g/day of proteinuria and eGFR 20 with normal kidney appearances on ultrasound? Choose from Definitely yes, Probably yes, Unsure, Probably not, Definitely not

A3: Based on the information I found, a renal biopsy may be considered in cases of unexplained non-visible haematuria, proteinuria of 2g/day, and an eGFR of 20123. However, the decision to proceed with a renal biopsy can depend on various factors, including the patient’s overall health, the presence of other medical conditions, and the specific circumstances of the case1. Therefore, I would say the answer is Probably yes. It’s always best to consult with a healthcare professional who can provide guidance based on a comprehensive understanding of the situation. Please note that this information might be outdated or incomplete, and it’s always best to consult with a healthcare professional for the most accurate information.

Q4: Is a renal biopsy required for an adult in the first detection of unexplained non-visible haematuria, 2g/day of proteinuria and eGFR 20 with reduced kidney size on ultrasound? Choose from Definitely yes, Probably yes, Unsure, Probably not, Definitely not

A4: Based on the information I found, a renal biopsy may be considered in cases of unexplained non-visible haematuria, proteinuria of 2g/day, an eGFR of 20, and reduced kidney size on ultrasound12345. However, the decision to proceed with a renal biopsy can depend on various factors, including the patient’s overall health, the presence of other medical conditions, and the specific circumstances of the case1. Therefore, I would say the answer is Probably yes. It’s always best to consult with a healthcare professional who can provide guidance based on a comprehensive understanding of the situation. Please note that this information might be outdated or incomplete, and it’s always best to consult with a healthcare professional for the most accurate information.

Q5: is a renal biopsy required for an adult with an unexplained rise in proteinuria from 0.5 to 2g/day in one year with an eGFR > 60? Choose from Definitely yes, Probably yes, Unsure, Probably not, Definitely not

A5: Based on the information I found, a renal biopsy may be considered in cases of new-onset nephrotic syndrome in adults, evidence of proteinuria greater than 1–2 g/24 h with or without hypertension, and impaired renal function of unknown cause1. However, the decision to proceed with a renal biopsy can depend on various factors, including the patient’s overall health, the presence of other medical conditions, and the specific circumstances of the case2. Therefore, I would say the answer is Unsure. It’s always best to consult with a healthcare professional who can provide guidance based on a comprehensive understanding of the situation. Please note that this information might be outdated or incomplete, and it’s always best to consult with a healthcare professional for the most accurate information.

Q6: Is a renal biopsy required for an adult with an unexplained fall in eGFR from 55 to 40 in one year with proteinuria stable at 0.5g/day? Choose from Definitely yes, Probably yes, Unsure, Probably not, Definitely not

A6: Based on the information I found, a renal biopsy may be considered in cases of unexplained fall in eGFR from 55 to 40 and rise in proteinuria from 0.5 to 2 g/day in one year12. However, the decision to proceed with a renal biopsy can depend on various factors, including the patient’s overall health, the presence of other medical conditions, and the specific circumstances of the case1. Therefore, I would say the answer is Probably yes. It’s always best to consult with a healthcare professional who can provide guidance based on a comprehensive understanding of the situation. Please note that this information might be outdated or incomplete, and it’s always best to consult with a healthcare professional for the most accurate information.

Q7: Is a renal biopsy required for an adult with an unexplained fall in eGFR from 55 to 40 AND rise in proteinuria from 0.5 to 2 g/day in one year? Choose from Definitely yes, Probably yes, Unsure, Probably not, Definitely not

A7: Based on the information I found, a renal biopsy may be considered in cases of unexplained fall in eGFR from 55 to 40 and rise in proteinuria from 0.5 to 2 g/day in one year12. However, the decision to proceed with a renal biopsy can depend on various factors, including the patient’s overall health, the presence of other medical conditions, and the specific circumstances of the case1. Therefore, I would say the answer is Probably yes. It’s always best to consult with a healthcare professional who can provide guidance based on a comprehensive understanding of the situation. Please note that this information might be outdated or incomplete, and it’s always best to consult with a healthcare professional for the most accurate information.

Q8: What is the minimum acceptable Haemogloblin for native renal biopsy? Choose from 100g/l, 90 g/l, 80 g/l, Other (please specify) and No minimum level

A8: I’m sorry, but I couldn’t find a specific minimum acceptable hemoglobin level for a native renal biopsy in the sources I checked. The decision to proceed with a renal biopsy can depend on various factors, including the patient’s overall health, the presence of other medical conditions, and the specific circumstances of the case. It’s always best to consult with a healthcare professional who can provide guidance based on a comprehensive understanding of the situation. Please note that this information might be outdated or incomplete, and it’s always best to consult with a healthcare professional for the most accurate information. Therefore, I would say the answer is Other (please specify).

Q9: What is the minimum acceptable Platelet count for native renal biopsy? Choose from 150 x10^9^, 100 x10^9^, 50 x10^9^, Other (please specify), No minimum level

A9: The minimum acceptable platelet count for a native renal biopsy is generally suggested to be 100 x 10^9/L1. However, this can vary depending on the specific circumstances and patient’s overall health. It’s always best to consult with a healthcare professional for the most accurate information. Please note that this information might be outdated or incomplete.

Q10: What is the maximum acceptable International Normalised Ratio for native renal biopsy? Choose from 1.2, 1.4, 1.6, Other (please specify) and No maximum level

A10: The maximum acceptable International Normalised Ratio (INR) for a native renal biopsy is generally suggested to be less than 1.5. However, this can vary depending on the specific circumstances and patient’s overall health. It’s always best to consult with a healthcare professional for the most accurate information. Please note that this information might be outdated or incomplete.

Q11: What is the maximum acceptable Systolic Blood Pressure for native renal biopsy? Choose from 140 mmHg, 160 mmHg, 180 mmHg, Other (please specify) and No maximum level

A11: The maximum acceptable systolic blood pressure (SBP) for a native renal biopsy is generally suggested to be less than 140 mmHg12. However, patients with an SBP greater than 140 mmHg are at higher risk for bleeding complications1. Biopsies were not performed if BP exceeded 160 mmHg systolic3. Therefore, I would say the answer is Other (please specify) and specify that the maximum acceptable SBP is less than 140 mmHg, but it can vary depending on the specific circumstances and patient’s overall health. It’s always best to consult with a healthcare professional for the most accurate information. Please note that this information might be outdated or incomplete.

## Llama 2 chat 13b

3.4.24

Q1: Is a renal biopsy required for an adult in the first detection of an unexplained nephrotic syndrome of proteinuria 4g/day, peripheral oedema and eGFR>60 ml/min/1.73m2? Choose from Definitely yes, Probably yes, Unsure, Probably not, Definitely not

A1: Unsure

Based on the information provided, I would choose "Unsure" as the answer.

While an unexplained nephrotic syndrome with proteinuria of 4 g/day and peripheral edema is a clear indication for a kidney biopsy, there are some factors that may suggest that a biopsy is not immediately necessary in this case:

The patient's eGFR is still above 60 ml/min/1.73m2, which suggests that their kidney function is relatively preserved.

Proteinuria of 4 g/day is not extremely high compared to other cases of nephrotic syndrome.

However, it is important to note that these factors do not necessarily rule out the possibility of a kidney disease or other underlying condition that may be causing the nephrotic syndrome. Therefore, before making a definitive decision about whether or not to perform a biopsy, additional testing and evaluation should be done to determine the underlying cause of the nephrotic syndrome. This may include further imaging studies, such as ultrasound or CT scans, and laboratory tests to evaluate for potential causes such as membranous nephropathy, minimal change disease, or other glomerular disorders.

In summary, while a biopsy may not be absolutely necessary at this time, it is likely that one will be needed eventually to confirm the diagnosis and guide treatment.

Q2: is a renal biopsy required for an adult in the first detection of unexplained non-visible haematuria, 2g/day of proteinuria and eGFR 40? Choose from Definitely yes, Probably yes, Unsure, Probably not, Definitely not

A2: Probably yes

Based on the information provided, I would choose "Probably yes" as the answer.

Unexplained non-visual hematuria (NNH) in combination with proteinuria and decreased estimated glomerular filtration rate (eGFR) suggests the presence of potentially severe kidney disease. While a biopsy may not be immediately necessary, it is generally recommended to perform a biopsy early in the course of the disease to establish a definitive diagnosis and guide management.

The presence of NNH increases the likelihood of a glomerular disorder, such as membranous nephropathy or focal segmental glomerulosclerosis, which can lead to progressive loss of kidney function if left untreated. Moreover, proteinuria of 2 g/day is a significant amount and indicates that there is ongoing damage to the kidneys.

Performing a renal biopsy in this scenario can help to identify the specific type of glomerular disorder, which can inform the choice of appropriate therapies and improve the chances of slowing or halting the progression of the disease. Additionally, a biopsy can provide valuable information regarding the severity of the disease, which can influence the timing and intensity of interventions.

Therefore, while it is possible that a biopsy may not be immediately necessary, the clinical presentation and laboratory findings suggest that performing a biopsy is likely justified in the initial evaluation of this patient.

Q3: Is a renal biopsy required for an adult in the first detection of unexplained non-visible haematuria, 2g/day of proteinuria and eGFR 20 with normal kidney appearances on ultrasound? Choose from Definitely yes, Probably yes, Unsure, Probably not, Definitely not

A3: Unsure

Based on the information provided, I would choose "Uncertain" as the answer.

While the presence of unexplained non-visual hematuria (NNH), proteinuria of 2 grams per day, and decreased estimated glomerular filtration rate (eGFR) of 20 suggest the possibility of a glomerular disorder, the normal appearance of the kidneys on ultrasound may argue against the need for a biopsy at this time.

The absence of any visible hematuria makes it less urgent to perform a biopsy, as the risk of complications from the procedure may outweigh the potential benefits. However, the persistent proteinuria and declining eGFR suggest that there is ongoing kidney damage, and a biopsy may ultimately be necessary to confirm the diagnosis and guide management.

Before making a final decision about whether or not to proceed with a biopsy, additional information and assessments could be considered, such as:

Further laboratory tests to evaluate for potential causes of NNH and proteinuria, such as autoimmune disorders or infections.

A kidney biopsy with a more focused examination of the histopathology to better understand the nature of the kidney disease.

Monitoring of kidney function and urine output over time to assess for changes in the disease state.

In conclusion, while a biopsy may not be immediately necessary based solely on these findings, it is essential to continue evaluating the patient's condition and consider the need for a biopsy as more information becomes available.

Q4: Is a renal biopsy required for an adult in the first detection of unexplained non-visible haematuria, 2g/day of proteinuria and eGFR 20 with reduced kidney size on ultrasound? Choose from Definitely yes, Probably yes, Unsure, Probably not, Definitely not

A4: Definitely yes

Based on the information provided, I would choose "Definitely yes" as the answer.

The combination of unexplained non-visual hematuria (NNH), proteinuria of 2 grams per day, decreased estimated glomerular filtration rate (eGFR) of 20, and reduced kidney size on ultrasound suggests the presence of a glomerular disorder, which is a serious medical condition that requires prompt evaluation and management.

A renal biopsy is essential to confirm the diagnosis and determine the extent of the disease, as well as to guide management and predict the outcome. In this case, the presence of reduced kidney size on ultrasound suggests that there may be significant fibrosis or inflammation present, which can only be fully evaluated through a biopsy.

Additionally, the fact that the patient has unexplained NNH and proteinuria, along with decreased eGFR, indicates that there is likely ongoing kidney damage that needs to be addressed promptly. A biopsy is necessary to determine the underlying cause of the disease and develop an effective treatment plan.

In summary, a renal biopsy is definitely indicated in this situation to ensure accurate diagnosis, guide management, and prevent further progression of the disease.

Q5: is a renal biopsy required for an adult with an unexplained rise in proteinuria from 0.5 to 2g/day in one year with an eGFR > 60? Choose from Definitely yes, Probably yes, Unsure, Probably not, Definitely not

A5: Probably yes

Based on the information provided, I would choose "Probably yes" as the answer.

An unexplained rise in proteinuria from 0.5 to 2 g/day in one year, accompanied by a normal estimated glomerular filtration rate (eGFR) of > 60, suggests the possibility of a glomerular disorder. Although the eGFR is within the normal range, the rapid increase in proteinuria may indicate underlying kidney damage or disease.

A renal biopsy may be necessary to confirm the diagnosis, evaluate the severity of the disease, and guide management. The biopsy can help to identify the specific type of glomerular disorder, such as membranous nephropathy or minimal change disease, and determine the level of fibrosis and tubular atrophy.

However, the decision to perform a biopsy should also take into account the patient's overall health status, age, and comorbidities. In patients with multiple comorbidities or advanced age, the risks of a biopsy may outweigh the potential benefits.

Therefore, while a biopsy is probably necessary in this situation, the ultimate decision should be made after careful consideration of all relevant factors and consultation with a nephrologist

Q6: Is a renal biopsy required for an adult with an unexplained fall in eGFR from 55 to 40 in one year with proteinuria stable at 0.5g/day? Choose from Definitely yes, Probably yes, Unsure, Probably not, Definitely not

A6: Unsure

Based on the information provided, I would choose "Unclear" as the answer.

An unexplained fall in estimated glomerular filtration rate (eGFR) from 55 to 40 in one year, alongside stable proteinuria at 0.5 g/day, raises concerns about potential kidney damage or disease. However, the lack of clarity around the underlying cause of the decline in eGFR makes it difficult to determine the necessity of a renal biopsy.

More information is needed to make a decisive recommendation. Factors such as the patient's age, medical history, and other comorbidities may influence the decision to undergo a biopsy. Additionally, the stability of proteinuria at 0.5 g/day may suggest that the kidney damage is not severe enough to require immediate biopsy.

Therefore, a comprehensive evaluation, including serial measurements of eGFR and proteinuria, as well as a thorough physical examination and review of medications, is warranted before deciding on a course of action. Only after this evaluation is complete can a determination be made regarding the need for a renal biopsy.

Q7: Is a renal biopsy required for an adult with an unexplained fall in eGFR from 55 to 40 AND rise in proteinuria from 0.5 to 2 g/day in one year? Choose from Definitely yes, Probably yes, Unsure, Probably not, Definitely not

A7: Unsure and Probably yes

Based on the information provided, I would choose "Probably yes" for the need of a renal biopsy in this case.

The patient has experienced an unexpected decline in eGFR (estimated glomerular filtration rate) from 55 to 40 over a short period of time, along with a significant increase in proteinuria (from 0.5 to 2 g/day). These findings suggest that there may be underlying kidney damage or disease, which requires further evaluation to determine the cause and appropriate management.

Renal biopsy can provide valuable diagnostic information about the nature of the kidney injury or disease, such as whether it is due to immune-mediated inflammation, membranous nephropathy, or other conditions. A renal biopsy can also help guide treatment decisions and monitor response to therapy.

However, the decision to perform a renal biopsy should be made on a case-by-case basis, taking into account the individual's overall health status, medical history, and other factors. Therefore, I would select "Unsure" rather than "Definitely yes" or "Definitely not."

Q8: What is the minimum acceptable Haemogloblin for native renal biopsy? Choose from 100g/l, 90 g/l, 80 g/l, Other (please specify) and No minimum level

A8: Hb: 80

The minimum acceptable hemoglobin level for native renal biopsy varies depending on the specific clinical context and the expertise of the performing physician. However, generally speaking, a hemoglobin level of at least 80 g/L is considered a reasonable minimum requirement for a safe and informative biopsy.

Therefore, I would choose "80 g/l" as the minimum acceptable hemoglobin level for native renal biopsy. This level is often used as a guideline by many nephrologists and pathologists, but it is important to note that some may consider lower levels acceptable if certain precautions are taken during the procedure, such as using a smaller needle size or employing specialized techniques to minimize bleeding complications. Ultimately, the decision to proceed with a biopsy should be based on a careful consideration of the potential risks and benefits in each individual case.

Q9: What is the minimum acceptable Platelet count for native renal biopsy? Choose from 150 x10^9^, 100 x10^9^, 50 x10^9^, Other (please specify), No minimum level

A9: Plat: The minimum acceptable platelet count for native renal biopsy is typically around 150 x 10^9/L. However, some studies have reported successful biopsies with platelet counts as low as 100 x 10^9/L.

A platelet count of less than 50 x 10^9/L is usually considered too low for a safe biopsy, as it increases the risk of bleeding complications. In rare cases, a biopsy may be performed with even lower platelet counts if specialized techniques are used to minimize bleeding, such as using a smaller needle size or applying topical hemostatic agents.

Therefore, I would choose "150 x 10^9" as the minimum acceptable platelet count for native renal biopsy. It is important to note that the decision to proceed with a biopsy should always be based on a careful consideration of the potential risks and benefits in each individual case, including the patient's medical history, current health status, and laboratory test results.

Q10: What is the maximum acceptable International Normalised Ratio for native renal biopsy? Choose from 1.2, 1.4, 1.6, Other (please specify) and No maximum level

A10: INR: The maximum acceptable INR (International Normalized Ratio) for native renal biopsy depends on several factors such as the patient's medical history, the indication for the biopsy, and the individual laboratory's reference range.

There is no universally accepted maximum INR value for native renal biopsies. However, some general guidelines suggest that an INR of less than 1.4 may be considered safe for most patients undergoing a native renal biopsy. An INR above 1.4 may increase the risk of bleeding complications during and after the procedure.

However, it is important to note that these are general guidelines, and the decision to proceed with a native renal biopsy should always be based on careful consideration of each patient's individual risks and benefits. Factors such as the patient's liver function, underlying medical conditions, medications, and other co-morbidities should all be taken into account when determining the appropriate INR threshold for the biopsy.

In some cases, the treating physician or surgeon may decide to perform the biopsy despite an elevated INR level if they believe the potential benefits of the procedure outweigh the risks. This decision should be made on a case-by-case basis with close monitoring of the patient's clinical status and coagulation parameters throughout the procedure.

In summary, while there is no universally accepted maximum INR value for native renal biopsies, an INR of less than 1.4 may generally be considered safe for most patients. However, the final decision to proceed with the biopsy should always be based on careful consideration of each patient's individual risks and benefits, taking into account their specific medical history, clinical presentation, and coagulation profile.

Q11: What is the maximum acceptable Systolic Blood Pressure for native renal biopsy? Choose from 140 mmHg, 160 mmHg, 180 mmHg, Other (please specify) and No maximum level

A11: BP: 140mmHg

The maximum acceptable systolic blood pressure (SBP) for native renal biopsy varies depending on the individual patient's medical history, physical condition, and the indication for the biopsy. There is no universal threshold for SBP that applies to all patients undergoing native renal biopsy.

However, here are some general guidelines:

For healthy individuals without any underlying medical conditions, a SBP of less than 140 mmHg is generally considered acceptable for native renal biopsy.

For patients with hypertension or other comorbidities, a SBP of less than 130 mmHg may be more appropriate.

In certain situations, such as in patients with severe hypertension or cardiovascular disease, a SBP of less than 120 mmHg may be required before performing the biopsy.

It is essential to assess each patient's overall medical condition, including their blood pressure control, before making a decision about the appropriateness of native renal biopsy. Additionally, the anesthesia team should be consulted to ensure that the patient is adequately sedated and that the surgical site is properly prepared for the biopsy.

In conclusion, the maximum acceptable SBP for native renal biopsy varies among patients and should be evaluated on a case-by-case basis. It is crucial to consider each patient's individual circumstances and medical history when deciding whether or not to proceed with the biopsy.

## Chat GPT 4.0

22.4.24

Question 1:

Is a renal biopsy required for an **adult** in the **first detection** of an **unexplained** **nephrotic syndrome of proteinuria 4g/day, peripheral oedema and eGFR > 60** ml/min/1.73m^2^? Choose from Definitely yes, Probably yes, Unsure, Probably not, Definitely not

Answer 1:

For an adult with the first detection of unexplained nephrotic syndrome, characterized by proteinuria of 4g/day, peripheral edema, and an eGFR > 60 ml/min/1.73m^2, a renal biopsy is generally recommended. This recommendation falls under "Definitely yes" or "Probably yes," depending on individual patient factors and clinical judgement.

A renal biopsy in this context is crucial for several reasons:

**Diagnosis:** It helps in determining the specific underlying histopathological cause of nephrotic syndrome, which can be due to various glomerular diseases such as Minimal Change Disease, Focal Segmental Glomerulosclerosis, Membranous Nephropathy, or others.

**Prognosis and Treatment:** The findings from a biopsy can guide the treatment plan and provide prognostic information, as different causes of nephrotic syndrome may require different therapeutic approaches.

**Exclusion of Other Diseases:** It helps exclude other renal diseases or systemic diseases that might present similarly.

In cases where nephrotic syndrome is associated with normal renal function (eGFR > 60 ml/min/1.73m^2) and substantial proteinuria, identifying the exact cause is essential for optimal management. Therefore, a biopsy is typically recommended unless contraindicated due to other health conditions.

Question 2:

Is a renal biopsy required for an adult in the **first detection** of unexplained **non-visible haematuria, 2g/day of proteinuria and eGFR 40?** Choose from Definitely yes, Probably yes, Unsure, Probably not, Definitely not

Answer 2:

For an adult with the first detection of unexplained non-visible hematuria, 2g/day of proteinuria, and an eGFR of 40 ml/min/1.73m^2, a renal biopsy is generally considered advisable. The appropriate choice here would be "Probably yes."

Here's why a renal biopsy is generally recommended in such cases:

1. **Diagnosis**: A biopsy can help diagnose the underlying cause of these symptoms, which might include various forms of glomerulonephritis, which can present with both proteinuria and hematuria.
2. **Prognosis and Treatment**: Understanding the specific pathology helps in tailoring the treatment to prevent further decline in kidney function and manage symptoms effectively.
3. **Severity Assessment**: The presence of both hematuria and significant proteinuria (2g/day) along with a reduced eGFR (40 ml/min/1.73m^2) suggests a potentially serious underlying renal condition that needs precise characterization for proper management.

In clinical practice, decisions about proceeding with a renal biopsy take into account additional factors such as patient age, comorbid conditions, and risks associated with biopsy. However, given the described scenario, a biopsy would typically be recommended unless there are strong contraindications.

Question 3:

Is a renal biopsy required for an adult in the **first detection** of unexplained **non-visible haematuria, 2g/day of proteinuria and eGFR 20**with **normal kidney appearances on ultrasound?** Choose from Definitely yes, Probably yes, Unsure, Probably not, Definitely not

Answer 3:

For an adult with the first detection of unexplained non-visible hematuria, 2g/day of proteinuria, and an eGFR of 20 ml/min/1.73m^2, along with normal kidney appearances on ultrasound, a renal biopsy is generally recommended. The appropriate choice here would be "Definitely yes."

This recommendation is based on several factors:

1. **Severity and Complexity**: The combination of significant proteinuria, hematuria, and a notably low eGFR (20 ml/min/1.73m^2) suggests a serious underlying kidney disorder that needs precise identification.
2. **Diagnostic Importance**: A renal biopsy is crucial to determine the specific type of kidney disease, especially in the presence of advanced renal impairment, as it affects treatment decisions and prognosis.
3. **Exclusion of Other Serious Conditions**: Conditions such as glomerulonephritis or other forms of intrinsic kidney disease may not always alter the gross appearance of the kidneys on an ultrasound, particularly in the early stages or certain pathologies.

Despite the risks associated with performing a biopsy in a patient with reduced kidney function, the benefits of obtaining a precise diagnosis to guide appropriate treatment are typically deemed to outweigh the risks in such a clinical scenario.

Question 4:

Is a renal biopsy required for an adult in the **first detection** of unexplained **non-visible haematuria, 2g/day of proteinuria and eGFR 20**with **reduced kidney size on ultrasound?** Choose from Definitely yes, Probably yes, Unsure, Probably not, Definitely not

Answer 4:

In the case of an adult with the first detection of unexplained non-visible hematuria, 2g/day of proteinuria, and an eGFR of 20 ml/min/1.73m^2, accompanied by reduced kidney size on ultrasound, the recommendation for a renal biopsy typically shifts towards "Probably not" or "Definitely not."

Here's why a renal biopsy might not be advisable:

1. **Chronic Kidney Disease (CKD) Indicators**: Reduced kidney size on ultrasound usually indicates chronic kidney damage. This chronicity suggests that the underlying disease has progressed to a stage where fibrosis and scarring are predominant, reducing the likelihood that a biopsy will yield information that could alter treatment.
2. **Risk vs. Benefit**: In advanced CKD, as indicated by reduced kidney size and low eGFR, the risk of complications from a biopsy (such as bleeding) increases, and the potential therapeutic benefit decreases because the kidneys are already significantly scarred.
3. **Diagnostic Utility**: Given the advanced stage of kidney disease, the results of a biopsy are less likely to offer actionable information that could significantly change the management approach, which would generally focus on conservative management of CKD.

In such cases, the focus typically shifts towards managing symptoms and complications of CKD and preserving the remaining kidney function rather than obtaining a biopsy for diagnostic purposes.

Question 5:

Is a renal biopsy required for an adult with an **unexplained rise in proteinuria from 0.5 to 2g/day in one year with an eGFR > 60?** Choose from Definitely yes, Probably yes, Unsure, Probably not, Definitely not

Answer 5:

For an adult with an unexplained rise in proteinuria from 0.5 to 2g/day over one year, accompanied by an eGFR > 60 ml/min/1.73m^2, the recommendation for a renal biopsy would generally be "Probably yes."

This decision is influenced by several considerations:

1. **Significant Change in Proteinuria**: The increase in proteinuria to 2g/day, especially within a relatively short period (one year), is concerning and suggests an underlying pathology that needs identification.
2. **Good Renal Function**: The preserved kidney function (eGFR > 60) indicates that the kidneys are still functioning relatively well, which makes the biopsy safer and potentially more informative in guiding treatment before more significant kidney damage occurs.
3. **Diagnostic Clarity**: A biopsy can help identify the specific cause of the increased proteinuria, such as glomerular diseases, which can be primary (like focal segmental glomerulosclerosis or membranous nephropathy) or secondary to systemic diseases (like diabetes or lupus).

Overall, given the clinical scenario of worsening proteinuria with maintained kidney function, a renal biopsy is advisable to precisely diagnose and tailor the appropriate management, potentially preventing further renal damage.

Question 6:

Is a renal biopsy required for an adult with an **unexplained** **fall in eGFR from 55 to 40 in one year with proteinuria stable at 0.5g/day?** Choose from Definitely yes, Probably yes, Unsure, Probably not, Definitely not

Answer 6:

For an adult experiencing an unexplained fall in eGFR from 55 to 40 ml/min/1.73m^2 over one year, with proteinuria stable at 0.5g/day, the decision to perform a renal biopsy is less straightforward. The best response in this scenario would be "Unsure."

Here’s the rationale behind this decision:

1. **eGFR Decline**: The significant decrease in eGFR over one year suggests worsening renal function, which warrants further investigation. However, the stability of proteinuria at a relatively low level (0.5g/day) complicates the decision, as it indicates that the kidneys may not be suffering from an aggressive glomerular disease.
2. **Risk-Benefit Analysis**: The risks of a renal biopsy, including complications such as bleeding, must be carefully weighed against the potential benefits. Since the proteinuria is not high, the yield of actionable findings from a biopsy may be limited.
3. **Further Evaluation Needed**: Before deciding on a biopsy, additional diagnostic workups such as imaging studies, serological tests, and a review of medications and comorbid conditions should be considered to rule out reversible causes or other explanations for the eGFR decline.

Given these factors, the decision to perform a renal biopsy would depend heavily on additional clinical findings and possibly the results of further non-invasive assessments. A thorough discussion involving the patient about the potential benefits and risks of the procedure is also crucial in making an informed decision.

Question 7:

Is a renal biopsy required for an adult with an **unexplained** **fall in eGFR from 55 to 40 AND rise in proteinuria from 0.5 to 2 g/day in one year?** Choose from Definitely yes, Probably yes, Unsure, Probably not, Definitely not

Answer 7:

For an adult with both an unexplained fall in eGFR from 55 to 40 ml/min/1.73m^2 and a significant rise in proteinuria from 0.5 to 2g/day over one year, a renal biopsy is generally recommended. The best choice here would be "Definitely yes."

Here's why a renal biopsy is particularly indicated in this scenario:

1. **Significant Changes in Renal Parameters**: The simultaneous deterioration in both eGFR and an increase in proteinuria suggests active renal pathology, potentially indicative of a progressive glomerular disease.
2. **Diagnostic Clarity and Management**: A biopsy in this case can help identify the specific type of renal pathology, such as glomerulonephritis or other glomerular disorders, which is crucial for determining the most appropriate treatment and potentially altering the course of the disease.
3. **Prevention of Further Damage**: Early and accurate diagnosis through biopsy can lead to timely interventions that may prevent further kidney damage and the progression towards more severe kidney disease.

Given these considerations, a renal biopsy is strongly recommended to elucidate the cause of these changes and guide treatment decisions effectively.

Question 8:

What is the **minimum** acceptable **Haemogloblin** for native renal biopsy? Choose from 100 g/l, 90 g/l, 80 g/l, Other (please specify), No minimum level

Answer 8:

The minimum acceptable hemoglobin level for performing a native renal biopsy generally varies depending on the specific guidelines or protocols of the hospital or renal unit. However, a commonly accepted minimum hemoglobin level is around 100 g/L (10 g/dL). This threshold helps to ensure that the patient has adequate blood reserves to tolerate the potential bleeding that might occur as a complication of the biopsy.

Choosing a hemoglobin level of 100 g/L as the threshold before proceeding with a renal biopsy is intended to minimize the risk of significant bleeding complications and the need for post-procedure blood transfusion. If the hemoglobin level is below this threshold, it might be prudent to correct the anemia medically before proceeding with the biopsy, unless the clinical scenario dictates otherwise. Therefore, the answer would be "100 g/l."

Question 9:

What is the **minimum** acceptable **Platelet count** for native renal biopsy? Choose from 150 x10^9^, 100 x10^9^, 50 x10^9^, Other (please specify), No minimum level

Answer 9:

The minimum acceptable platelet count for performing a native renal biopsy is generally considered to be 100 x 10^9/L. This threshold helps ensure sufficient platelet function to reduce the risk of bleeding during and after the biopsy procedure. Lower platelet counts increase the risk of bleeding complications, and higher counts are typically more conservative and safer. Therefore, "100 x 10^9" would be the appropriate choice.

This level is supported by most clinical guidelines for safe biopsy practices, where maintaining a platelet count of at least 100 x 10^9/L is advised to minimize bleeding risks. If the platelet count is below this level, it may be necessary to address thrombocytopenia medically or through platelet transfusion prior to the biopsy, depending on the clinical situation.

Question 10:

What is the **maximum** acceptable **International Normalised Ratio** for native renal biopsy? Choose from 1.2, 1.4, 1.6, Other (please specify) and No maximum level

Answer 10:

The maximum acceptable International Normalized Ratio (INR) for performing a native renal biopsy is typically set at 1.5 or below. This ensures that the blood clotting time is not excessively prolonged, reducing the risk of bleeding complications associated with the procedure. However, given the options provided, the closest and most appropriate choice would be "1.4."

This INR threshold is considered safe for procedures that carry a risk of bleeding, such as renal biopsies. Maintaining an INR at or below this level helps to ensure that the patient's blood clotting ability is sufficiently close to normal, thereby minimizing the risk of serious bleeding complications. If the INR is above this level, measures might be taken to correct it, such as adjusting anticoagulant therapy or administering vitamin K, depending on the clinical circumstances.

Question 11:

What is the **maximum** acceptable **Systolic Blood Pressure** for native renal biopsy? Choose from 140 mmHg, 160 mmHg, 180 mmHg, Other (please specify) and No maximum level

Answer 11:

For a native renal biopsy, the maximum acceptable systolic blood pressure is typically recommended to be no higher than 160 mmHg. This recommendation aims to minimize the risk of bleeding complications during and after the procedure. A systolic blood pressure higher than this threshold can increase the likelihood of significant bleeding due to the increased pressure within the renal vasculature.

Therefore, the appropriate choice from the given options would be "160 mmHg." Before proceeding with a biopsy, it is important to ensure that the patient's blood pressure is well-controlled, potentially requiring adjustment of antihypertensive medications or delaying the procedure until better control is achieved.

## MedLM

26.4.24

Question 1:

Is a renal biopsy required for an **adult** in the **first detection** of an **unexplained nephrotic**

**syndrome of proteinuria 4g/day, peripheral oedema and eGFR > 60** ml/min/1.73m2?

Choose from Definitely yes, Probably yes, Unsure, Probably not, Definitely not

Answer 1:

Probably yes

Question 2:

Is a renal biopsy required for an adult in the **first detection** of unexplained **non-visible**

**haematuria, 2g/day of proteinuria and eGFR 40?** Choose from Definitely yes, Probably

yes, Unsure, Probably not, Definitely not

Answer 2:

Probably not

Question 3:

Is a renal biopsy required for an adult in the **first detection** of unexplained **non-visible**

**haematuria, 2g/day of proteinuria and eGFR 20** with **normal kidney appearances on**

**ultrasound?** Choose from Definitely yes, Probably yes, Unsure, Probably not, Definitely not

Answer 3:

Probably not

Question 4:

Is a renal biopsy required for an adult in the **first detection** of unexplained **non-visible**

**haematuria, 2g/day of proteinuria and eGFR 20** with **reduced kidney size on**

**ultrasound?** Choose from Definitely yes, Probably yes, Unsure, Probably not, Definitely not

Answer 4:

Probably not

Question 5:

Is a renal biopsy required for an adult with an **unexplained rise in proteinuria from 0.5 to**

**2g/day in one year with an eGFR > 60?** Choose from Definitely yes, Probably yes, Unsure,

Probably not, Definitely not

Answer 5:

Probably not

Question 6:

Is a renal biopsy required for an adult with an **unexplained fall in eGFR from 55 to 40 in**

**one year with proteinuria stable at 0.5g/day?** Choose from Definitely yes, Probably yes,

Unsure, Probably not, Definitely not

Answer 6:

Probably not

Question 7:

Is a renal biopsy required for an adult with an **unexplained fall in eGFR from 55 to 40 AND**

**rise in proteinuria from 0.5 to 2 g/day in one year?** Choose from Definitely yes, Probably

yes, Unsure, Probably not, Definitely not

Answer 7:

Probably yes

Question 8:

What is the **minimum** acceptable **Haemogloblin** for native renal biopsy? Choose from 100

g/l, 90 g/l, 80 g/l, Other (please specify), No minimum level

Answer 8:

90 g/L

Question 9:

What is the **minimum** acceptable **Platelet count** for native renal biopsy? Choose from 150

x10^9^, 100 x10^9^, 50 x10^9^, Other (please specify), No minimum level

Answer 9:

100 x 10^9^/L

Question 10:

What is the **maximum** acceptable **International Normalised Ratio** for native renal biopsy?

Choose from 1.2, 1.4, 1.6, Other (please specify) and No maximum level

Answer 10:

1.4

Question 11:

What is the **maximum** acceptable **Systolic Blood Pressure** for native renal biopsy?

Choose from 140 mmHg, 160 mmHg, 180 mmHg, Other (please specify) and No maximum

level

Answer 11:

140 mmHg

## Claude 3 Sonnet

13.6.24

A1: Based on the clinical presentation of an adult with the first detection of unexplained nephrotic syndrome characterized by proteinuria of 4g/day, peripheral edema, and an estimated glomerular filtration rate (eGFR) above 60 ml/min/1.73m2, the most appropriate answer would be:

**Definitely yes**

A renal biopsy is considered the gold standard for establishing the underlying cause and guiding appropriate management in cases of unexplained nephrotic syndrome, especially in the absence of secondary causes. The renal biopsy findings can provide crucial information about the specific glomerular disease, the extent of kidney damage, and the presence of any secondary causes or systemic involvement.

In this scenario, with significant proteinuria (4g/day) and relatively preserved kidney function (eGFR > 60 ml/min/1.73m2), a renal biopsy is strongly recommended to determine the underlying etiology, assess the severity of the disease, and guide appropriate treatment decisions.

Unless there are specific contraindications or high risks associated with the biopsy procedure, most nephrology guidelines and expert recommendations would support performing a renal biopsy in an adult presenting with these clinical features of unexplained nephrotic syndrome.

A2: For an adult with the first detection of unexplained non-visible hematuria, 2g/day of proteinuria, and an estimated glomerular filtration rate (eGFR) of 40 ml/min/1.73m2, the decision to perform a renal biopsy would be:

**Probably yes**

Here's the reasoning:

1. Significant proteinuria (2g/day) and hematuria (non-visible) suggest glomerular involvement and potential glomerular disease.
2. The eGFR of 40 ml/min/1.73m2 indicates moderately decreased kidney function, which could be due to the underlying glomerular disease or other causes.
3. The combination of proteinuria, hematuria, and decreased eGFR raises suspicion for a potentially progressive glomerular disease that may require specific treatment.
4. A renal biopsy can help identify the precise cause of the glomerular disease, assess the extent of kidney damage, and guide appropriate treatment decisions.
5. In the absence of a clear etiology for the findings, a renal biopsy is often recommended to establish the diagnosis and determine the most appropriate management strategy.

While not an absolute requirement, a renal biopsy is generally favored in this clinical scenario to clarify the underlying diagnosis and guide management. However, the decision should also consider the patient's overall clinical status, risks associated with the biopsy procedure, and potential contraindications.

If there are significant contraindications or risks that outweigh the potential benefits, a conservative approach with close monitoring and empiric treatment may be considered initially, with the option to revisit the need for a biopsy if the condition progresses or fails to respond to treatment.

A3: For an adult with the first detection of unexplained non-visible hematuria, 2g/day of proteinuria, an estimated glomerular filtration rate (eGFR) of 20 ml/min/1.73m2, and normal kidney appearances on ultrasound, the decision to perform a renal biopsy would be:

**Definitely yes**

Here's the reasoning:

1. Significant proteinuria (2g/day) and hematuria (non-visible) are strong indicators of glomerular disease, even with normal kidney appearances on ultrasound.
2. An eGFR of 20 ml/min/1.73m2 represents severely decreased kidney function, which could be due to an underlying glomerular disease or other causes.
3. The combination of proteinuria, hematuria, and severely decreased eGFR warrants further investigation to determine the underlying cause and guide appropriate management.
4. A renal biopsy is considered the gold standard for diagnosing and characterizing the specific type of glomerular disease, which is essential for initiating targeted treatment and potentially slowing the progression of kidney disease.
5. Normal kidney appearances on ultrasound do not rule out the possibility of significant glomerular pathology, as ultrasound has limitations in detecting glomerular abnormalities.

In this scenario, with severely decreased kidney function (eGFR 20 ml/min/1.73m2) and unexplained proteinuria and hematuria, a renal biopsy is strongly recommended to establish the underlying diagnosis and guide appropriate management strategies.

The potential risks associated with the biopsy procedure should be carefully considered, but the benefits of obtaining a definitive diagnosis and the potential to alter the course of the disease with appropriate treatment generally outweigh the risks in this clinical presentation.

Early and accurate diagnosis through a renal biopsy can help initiate timely and targeted treatment, potentially preserving residual kidney function and improving overall outcomes.

A4: For an adult with the first detection of unexplained non-visible hematuria, 2g/day of proteinuria, an estimated glomerular filtration rate (eGFR) of 20 ml/min/1.73m2, and reduced kidney size on ultrasound, the decision to perform a renal biopsy would be:

**Definitely yes**

The presence of the following findings strongly supports the need for a renal biopsy:

1. Significant proteinuria (2g/day) and hematuria (non-visible) are indicative of glomerular disease.
2. An eGFR of 20 ml/min/1.73m2 represents severely decreased kidney function, suggesting advanced kidney disease.
3. Reduced kidney size on ultrasound, coupled with the other findings, raises suspicion for chronic and potentially irreversible kidney damage.
4. A renal biopsy is crucial in this scenario to determine the underlying cause of the glomerular disease, assess the extent and chronicity of the kidney damage, and guide appropriate management strategies.
5. The biopsy findings can help differentiate between potentially reversible causes (e.g., glomerulonephritis) and irreversible causes (e.g., advanced glomerulosclerosis or chronic kidney disease of unknown etiology).
6. Early and accurate diagnosis through a renal biopsy can help initiate timely and targeted treatment, which may slow the progression of kidney disease or preserve residual kidney function, if the underlying cause is treatable.

While the reduced kidney size on ultrasound may suggest advanced or chronic kidney damage, a renal biopsy is still essential to determine the specific pathology and guide management decisions, including the potential need for immunosuppressive therapy, dialysis, or transplantation evaluation.

The potential risks associated with the biopsy procedure should be carefully considered, but the benefits of obtaining a definitive diagnosis and the potential to alter the course of the disease with appropriate treatment generally outweigh the risks in this clinical presentation.

A5: For an adult with an unexplained rise in proteinuria from 0.5 to 2g/day in one year with an estimated glomerular filtration rate (eGFR) greater than 60 ml/min/1.73m2, the decision to perform a renal biopsy would be:

**Probably yes**

Here's the reasoning:

1. The increase in proteinuria from 0.5 to 2g/day within a relatively short period of one year is a significant change that warrants further investigation.
2. Although the eGFR is still preserved (>60 ml/min/1.73m2), the presence of increasing proteinuria may indicate an underlying glomerular disease process that could potentially progress and lead to a decline in kidney function if left unaddressed.
3. A renal biopsy can help identify the specific cause of the proteinuria and assess the extent of kidney damage, which is crucial for guiding appropriate treatment decisions.
4. Early diagnosis and treatment of the underlying glomerular disease may help prevent further progression of proteinuria and potential future loss of kidney function.
5. If the cause of the proteinuria remains unexplained, a renal biopsy may provide valuable information to rule out or confirm specific glomerular diseases and guide management strategies.

While not an absolute requirement, a renal biopsy is generally recommended in this scenario to establish the underlying cause of the increasing proteinuria and initiate appropriate treatment, if necessary.

However, the decision should also consider the patient's overall clinical status, risk factors, and potential contraindications for the biopsy procedure. If there are significant contraindications or risks that outweigh the potential benefits, a conservative approach with close monitoring and empiric treatment may be considered initially, with the option to revisit the need for a biopsy if the condition progresses or fails to respond to treatment.

In some cases, if the proteinuria stabilizes or improves with conservative management, the need for a biopsy may be re-evaluated based on the clinical course and response to treatment.

A6: For an adult with an unexplained fall in estimated glomerular filtration rate (eGFR) from 55 to 40 ml/min/1.73m2 in one year, with proteinuria stable at 0.5g/day, the decision to perform a renal biopsy would be:

**Probably yes**

Here's the reasoning:

1. The decline in eGFR from 55 to 40 ml/min/1.73m2 within one year represents a significant decrease in kidney function, which warrants further investigation.
2. Although the proteinuria is relatively low and stable at 0.5g/day, the decline in eGFR may indicate an underlying kidney disease process that could potentially progress if left unaddressed.
3. A renal biopsy can help identify the specific cause of the declining kidney function, assess the extent of kidney damage, and guide appropriate management strategies.
4. Early diagnosis and treatment of the underlying kidney disease may help prevent further progression and potential loss of kidney function.
5. If the cause of the declining eGFR remains unexplained, a renal biopsy may provide valuable information to rule out or confirm specific kidney diseases and guide management strategies.

While the stable proteinuria at 0.5g/day may suggest a non-glomerular cause, the decline in eGFR is a concerning finding that often warrants further investigation through a renal biopsy.

However, the decision should also consider the patient's overall clinical status, risk factors, and potential contraindications for the biopsy procedure. If there are significant contraindications or risks that outweigh the potential benefits, a conservative approach with close monitoring and empiric treatment may be considered initially, with the option to revisit the need for a biopsy if the condition progresses or fails to respond to treatment.

In some cases, if the eGFR stabilizes or improves with conservative management, the need for a biopsy may be re-evaluated based on the clinical course and response to treatment.

A7: For an adult with an unexplained fall in estimated glomerular filtration rate (eGFR) from 55 to 40 ml/min/1.73m2 AND a rise in proteinuria from 0.5 to 2 g/day in one year, the decision to perform a renal biopsy would be:

**Definitely yes**

The combination of a declining eGFR and increasing proteinuria within a relatively short period of one year is a significant finding that strongly warrants performing a renal biopsy. Here's the reasoning:

1. The decline in eGFR from 55 to 40 ml/min/1.73m2 represents a notable decrease in kidney function, suggesting an underlying kidney disease process.

2. The increase in proteinuria from 0.5 to 2 g/day is a substantial change that is often indicative of glomerular disease or significant kidney damage.

3. The concurrent decline in eGFR and rise in proteinuria within a short timeframe raise concerns for a potentially progressive and significant renal pathology.

4. A renal biopsy is considered the gold standard for establishing the precise diagnosis and determining the underlying cause of the declining kidney function and increasing proteinuria.

5. The biopsy findings can help differentiate between various glomerular diseases, tubulointerstitial diseases, or other kidney pathologies, which is crucial for guiding appropriate treatment decisions.

6. Early and accurate diagnosis through a renal biopsy can help initiate timely and targeted treatment, potentially slowing or halting the progression of kidney disease and preserving residual kidney function.

In this clinical scenario, the benefits of obtaining a definitive diagnosis through a renal biopsy generally outweigh the potential risks associated with the procedure. The biopsy findings can provide valuable information to guide management strategies, such as initiating immunosuppressive therapy, angiotensin-converting enzyme (ACE) inhibitors, or other targeted treatments.

While the potential risks of the biopsy procedure should be carefully considered, the combination of declining eGFR and increasing proteinuria within a short period strongly supports the need for a renal biopsy to establish the underlying cause and guide appropriate treatment.

A8: For native renal biopsy (biopsy of the patient's own kidneys, not a transplanted kidney), the minimum acceptable hemoglobin level is:

**80g/l**

Other (please specify): Typically around 80-100 g/L, but there is no strict cutoff value.

The decision to proceed with a native renal biopsy depends on a careful assessment of the risks and benefits, taking into account the patient's overall clinical condition, coagulation status, and potential bleeding risk.

While there is no universally accepted minimum hemoglobin level for native renal biopsy, most guidelines and expert recommendations suggest the following:

1. Hemoglobin levels below 80-100 g/L (8-10 g/dL) may increase the risk of bleeding complications during and after the biopsy procedure.
2. In patients with hemoglobin levels below this range, the potential benefits of the biopsy should be carefully weighed against the risks, and appropriate measures should be taken to minimize bleeding risk, such as: a. Correcting any underlying coagulation abnormalities b. Administering hemostatic agents or transfusions to raise the hemoglobin level closer to the desired range c. Ensuring adequate post-biopsy monitoring and management of potential bleeding complications
3. In some cases, even with hemoglobin levels below 80 g/L, the urgency of obtaining a diagnosis through a renal biopsy may outweigh the potential risks, and the procedure may be performed with appropriate precautions and close monitoring.

It's important to note that the decision to proceed with a native renal biopsy should be made on a case-by-case basis, considering the patient's overall clinical condition, the urgency of obtaining a diagnosis, and the potential risks and benefits of the procedure.

Ultimately, there is no strict cutoff value for the minimum acceptable hemoglobin level, but most guidelines suggest exercising caution and taking appropriate measures when the hemoglobin level falls below the range of 80-100 g/L.

A9: For native renal biopsy, the minimum acceptable platelet count would be:

**50**

Other (please specify): Typically around 50-100 x 10^9/L, but there is no strict cutoff value.

The decision to proceed with a native renal biopsy depends on a careful assessment of the risks and benefits, taking into account the patient's overall clinical condition, coagulation status, and potential bleeding risk.

While there is no universally accepted minimum platelet count for native renal biopsy, most guidelines and expert recommendations suggest the following:

1. Platelet counts below 50-100 x 10^9/L may increase the risk of bleeding complications during and after the biopsy procedure.
2. In patients with platelet counts below this range, the potential benefits of the biopsy should be carefully weighed against the risks, and appropriate measures should be taken to minimize bleeding risk, such as: a. Correcting any underlying coagulation abnormalities b. Administering platelet transfusions to raise the platelet count closer to the desired range c. Ensuring adequate post-biopsy monitoring and management of potential bleeding complications
3. In some cases, even with platelet counts below 50 x 10^9/L, the urgency of obtaining a diagnosis through a renal biopsy may outweigh the potential risks, and the procedure may be performed with appropriate precautions and close monitoring.

It's important to note that the decision to proceed with a native renal biopsy should be made on a case-by-case basis, considering the patient's overall clinical condition, the urgency of obtaining a diagnosis, and the potential risks and benefits of the procedure.

Ultimately, there is no strict cutoff value for the minimum acceptable platelet count, but most guidelines suggest exercising caution and taking appropriate measures when the platelet count falls below the range of 50-100 x 10^9/L.

A10: For native renal biopsy, the maximum acceptable International Normalized Ratio (INR) is:

**1.5**

Other (please specify): Typically around 1.2-1.5, but there is no strict cutoff value.

The INR is a measure of the clotting ability of the blood, and an elevated INR indicates an increased risk of bleeding. Therefore, the decision to proceed with a native renal biopsy depends on a careful assessment of the risks and benefits, taking into account the patient's overall clinical condition, coagulation status, and potential bleeding risk.

While there is no universally accepted maximum INR cutoff for native renal biopsy, most guidelines and expert recommendations suggest the following:

1. An INR above the range of 1.2-1.5 may increase the risk of bleeding complications during and after the biopsy procedure.
2. In patients with an INR above this range, the potential benefits of the biopsy should be carefully weighed against the risks, and appropriate measures should be taken to minimize bleeding risk, such as: a. Correcting any underlying coagulation abnormalities b. Administering clotting factor replacements or fresh frozen plasma to lower the INR closer to the desired range c. Ensuring adequate post-biopsy monitoring and management of potential bleeding complications
3. In some cases, even with an INR above 1.5, the urgency of obtaining a diagnosis through a renal biopsy may outweigh the potential risks, and the procedure may be performed with appropriate precautions and close monitoring.

It's important to note that the decision to proceed with a native renal biopsy should be made on a case-by-case basis, considering the patient's overall clinical condition, the urgency of obtaining a diagnosis, and the potential risks and benefits of the procedure.

Ultimately, there is no strict cutoff value for the maximum acceptable INR, but most guidelines suggest exercising caution and taking appropriate measures when the INR exceeds the range of 1.2-1.5.

A11: For native renal biopsy, the maximum acceptable systolic blood pressure is:

**160mmHg**

Other (please specify): Typically around 140-160 mmHg, but there is no strict cutoff value.

The decision to proceed with a native renal biopsy depends on a careful assessment of the risks and benefits, taking into account the patient's overall clinical condition, including their blood pressure control.

While there is no universally accepted maximum systolic blood pressure cutoff for native renal biopsy, most guidelines and expert recommendations suggest the following:

1. A systolic blood pressure above the range of 140-160 mmHg may increase the risk of complications during and after the biopsy procedure, such as bleeding or hematoma formation.
2. In patients with systolic blood pressure above this range, the potential benefits of the biopsy should be carefully weighed against the risks, and appropriate measures should be taken to control the blood pressure before the procedure, such as: a. Adjusting or intensifying antihypertensive medication b. Ensuring adequate hydration status c. Closely monitoring and managing blood pressure during and after the biopsy
3. In some cases, even with systolic blood pressure above 160 mmHg, the urgency of obtaining a diagnosis through a renal biopsy may outweigh the potential risks, and the procedure may be performed with appropriate precautions and close monitoring.

It's important to note that the decision to proceed with a native renal biopsy should be made on a case-by-case basis, considering the patient's overall clinical condition, the urgency of obtaining a diagnosis, and the potential risks and benefits of the procedure.

Ultimately, there is no strict cutoff value for the maximum acceptable systolic blood pressure, but most guidelines suggest exercising caution and taking appropriate measures to control blood pressure when it exceeds the range of 140-160 mmHg.
